# Supplementary material for: Macropinocytosis mediates resistance to loss of glutamine transport in triple-negative breast cancer
Source: EMBO J. 2024 Oct 17;43(23):5857–82. doi: 10.1038/s44318-024-00271-6 (PMC11611898; doi:10.1038/s44318-024-00271-6)
Supplement: Supplementary file 1 — Appendix [file 44318_2024_271_MOESM1_ESM.pdf]

Appendix for

Macropinocytosis mediates resistance to loss of glutamine transport in triple-negative breast cancer

Table of Contents:

Appendix Figure S1 (page 2).

A

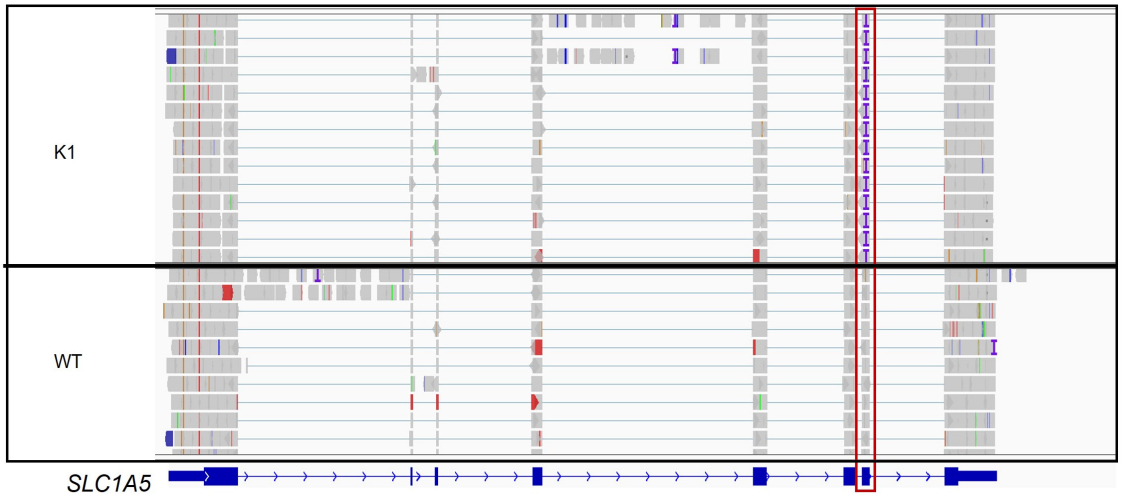

B

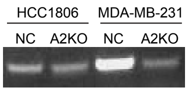

Cell lines	Sequence of *SLC1A5* Exon 7 gRNA site with insertion
HCC1806 NC	GGCCATCATCCTCGAAGCAGTCAACCTCCCGGTCGACCATATCTCCTT
HCC1806 A2KO	GGCCATCATCCTCGAAAGCAGTCAACCTCCCGGTCGACCATATCTCCTT
MDA-MB-231 NC	GGCCATCATCCTCGAAGCAGTCAACCTCCCGGTCGACCATATCTCCTTGATCCT
MDA-MB-231 A2KO	GGCCATCATCCTCGAAATCGCCGCGGTCCAGGACCCCTCGATCCTCCTTGCTGT

C

MVADPPRDSKGLAAAEPTANGGLALASIEDQGAAAGGYCGSRDQVRRCLRANLLVLLTVVAVVAGVALGL
GVSGAGGALALGPERLSAFVFPGELLLRLLRMIILPLVVCSLIGGAASLDPGALGRLGAWALLFFLVTTLLAS
ALGVGLALALQPGAASAAINASVGAAGSAENAPSKEVLDSFLDLARNIFPSNLVSAAFRSYSTTYEERNITG
TRVKVPVGQEVEGMNILGLVVFAIVFGVALRKLGPEGELLIRFFNSFNEATMVLVSWIMWYAPVGIMFLVAG
KIVEMEDVGLLFARLGKYILCCLLGHAIHGLLVLPLIYFLFTRKNPYRFLWGIVTPLATAFGTSSSSATLPLMM
KCVEENNGVAKHISRFILPIGATVNMDGAALFQCVAAVFIAQLSQQSLDFVKIITILVTATASSVGAAGIPAGGV
LTLAIILEAVNLPVDHISLILAVDWLVDRSCTVLNVEGDALGAGLLQNYVDRTESRSTEPELIQVKSELPLDPL
PVPTEEGNPLLKHYRGPAGDATVASEKESVM

ASCT2 peptides detected in Proteomics
Exon 4 coded amino acids
Exon 7 coded amino acids

Appendix Figure S1: CRISPR insertion site analyses and proteomics peptide sequences

(A) Integrative genomics viewer (IGV) plot from mRNA seq data for *SLC1A5* transcripts in HCC1806 WT and HCC1806 K1 cells aligned to Homo sapiens (human) genome assembly GRCh38 (hg38). Grey boxes in the IGV plot indicate exonic regions, with the red box marking exon 7 and 'I' indicates single nucleotide insertion in the knockout cell line. (B) PCR amplification of region spanning CRISPR guide RNA binding sites for exon 7 using forward primer 5' GGCATCTGCCTAACCTCC 3'and reverse primer 5' ATCCTCTTTCCCAGGGGTA 3' in polyclonal NC and A2KO cell lines. Sanger sequencing was performed for the amplified PCR product depicting exon 7 sequence (grey), guide RNA binding region (pink) and nucleotide insertion (green). (C) ASCT2 amino acid sequence showing proteomics peptides detected in HCC1806 WT cells (yellow highlight), amino acids coded by exon 4 are shown in blue text and those by exon 7 are shown in orange text.
