# Supplementary material for: Macropinocytosis mediates resistance to loss of glutamine transport in triple-negative breast cancer
Source: EMBO J. 2024 Oct 17;43(23):5857–82. doi: 10.1038/s44318-024-00271-6 (PMC11611898; doi:10.1038/s44318-024-00271-6)
Supplement: Supplementary file 5 — Source data Fig. 1 [file 44318_2024_271_MOESM5_ESM.zip › Figure 1/1J and K_FCS files/Sorting FCS files/20201215_1569_NC,CRA2#1,2 sort/1569 CRA2-2.pdf]

ACQUISITION DASHBOARD - SAMPLE RUNNING...

Unload Sample | Pause Sample | Flow Rate: 1 | Event Rate: 616

Total Events: 60,540
Processed Events: 99.84%
Elapsed Time: 00:01:45

Recording Criteria: 10,000 | Population: All Events | 0 — 10,000

Start Recording | ON Light | ON Agitation | Backflush

DATA SOURCES

Undo | Redo | Display Events 2,000 | Refresh Data

Live Data
60,540 events

1569 CRA2-2	5,696 events
12/15/2020 2:15:02 PM	
1569-CRA2-1	10,000 events
12/15/2020 2:13:06 PM	
1569-NC	5,148 events
12/15/2020 2:10:54 PM	

Update Compensation | Export FCS Files

POPULATION HIERARCHY

- All Events
  - Scatter
    - SSC Singlets
      - FSC Singlets
        - PE pos
          - PE Sort
        - PE Neg

THRESHOLD AND SCATTER SETUP • Doublet Discrimination

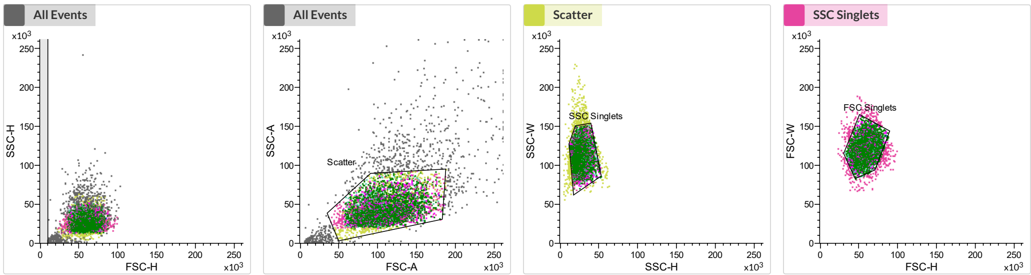

PLOTS

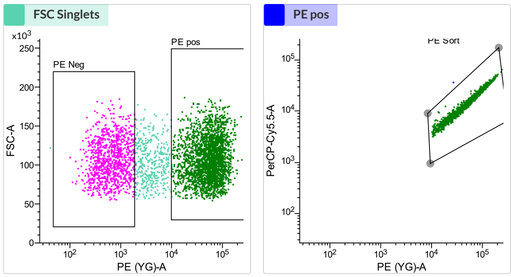

STATISTICS

Population	Events	% Parent	% Total	FSC-A Median	FSC-A %rCV	SSC-A Median	SSC-A %rCV
All Events	5,696		100.00 %	105629.27	33.79 %	43074.21	45.79 %
Scatter	4,753	83.44 %	83.44 %	105461.32	29.86 %	42155.55	37.23 %
SSC Singlets	4,294	90.34 %	75.39 %	105727.48	29.20 %	41852.23	34.51 %
FSC Singlets	3,921	91.31 %	68.84 %	105394.02	27.25 %	41639.56	33.85 %
PE pos	2,519	64.24 %	44.22 %	106267.08	26.99 %	41498.01	33.62 %
PE Sort	2,518	99.96 %	44.21 %	106248.59	27.00 %	41491.73	33.57 %
PE Neg	1,011	25.78 %	17.75 %	105795.99	27.76 %	40607.58	33.14 %
