# Supplementary material for: Macropinocytosis mediates resistance to loss of glutamine transport in triple-negative breast cancer
Source: EMBO J. 2024 Oct 17;43(23):5857–82. doi: 10.1038/s44318-024-00271-6 (PMC11611898; doi:10.1038/s44318-024-00271-6)
Supplement: Supplementary file 5 — Source data Fig. 1 [file 44318_2024_271_MOESM5_ESM.zip › Figure 1/1J and K_FCS files/Sorting FCS files/20201215_1569_NC,CRA2#1,2 sort/1569 CRA2-1.pdf]

ACQUISITION DASHBOARD - SAMPLE RUNNING...

Unload Sample | Pause Sample | Flow Rate: 1 | Event Rate: 652

Total Events: 53,085
Processed Events: 99.83%
Elapsed Time: 00:01:34

Recording Criteria: 10,000 | Population: All Events | 0 — 10,000

Start Recording | ON Light | ON Agitation | Backflush

DATA SOURCES

Undo | Redo | Display Events 2,000 | Refresh Data

Live Data
53,085 events

1569 CRA2-2	5,696 events
12/15/2020 2:15:02 PM	
1569-CRA2-1	10,000 events
12/15/2020 2:13:06 PM	
1569-NC	5,148 events
12/15/2020 2:10:54 PM	

Update Compensation | Export FCS Files

POPULATION HIERARCHY

- All Events
  - Scatter
    - SSC Singlets
      - FSC Singlets
        - PE pos
          - PE Sort
        - PE Neg

THRESHOLD AND SCATTER SETUP • Doublet Discrimination

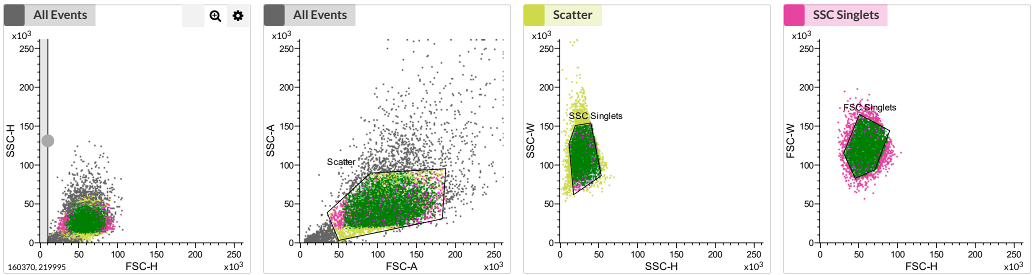

PLOTS

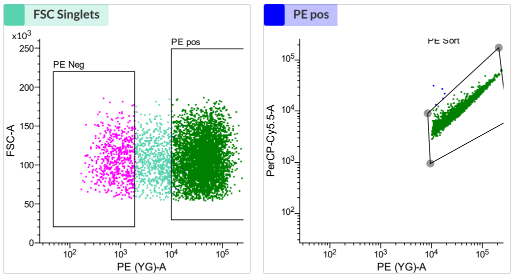

STATISTICS

Population	Events	% Parent	% Total	FSC-A Median	FSC-A %rCV	SSC-A Median	SSC-A %rCV
All Events	10,000		100.00 %	104079.90	35.99 %	43310.95	53.15 %
Scatter	7,796	77.96 %	77.96 %	106451.09	28.74 %	43302.92	40.34 %
SSC Singlets	6,931	88.90 %	69.31 %	107163.17	27.98 %	43123.38	36.61 %
FSC Singlets	6,180	89.16 %	61.80 %	106854.88	25.91 %	43138.71	36.23 %
PE pos	4,854	78.54 %	48.54 %	107110.13	26.14 %	42989.15	35.98 %
PE Sort	4,849	99.90 %	48.49 %	107112.85	26.12 %	42973.46	35.94 %
PE Neg	641	10.37 %	6.41 %	107509.36	25.60 %	41329.96	34.45 %
