# Supplementary material for: Macropinocytosis mediates resistance to loss of glutamine transport in triple-negative breast cancer
Source: EMBO J. 2024 Oct 17;43(23):5857–82. doi: 10.1038/s44318-024-00271-6 (PMC11611898; doi:10.1038/s44318-024-00271-6)
Supplement: Supplementary file 5 — Source data Fig. 1 [file 44318_2024_271_MOESM5_ESM.zip › Figure 1/1J and K_FCS files/Sorting FCS files/20201215_1569_NC,CRA2#1,2 sort/1569 NC.pdf]

ACQUISITION DASHBOARD - SAMPLE RUNNING...

Unload Sample | Pause Sample | Flow Rate: 1 | Event Rate: 812

Total Events: 44,084
Processed Events: 99.83%
Elapsed Time: 00:01:22

Recording Criteria: 10,000 | Population: All Events | 0 — 10,000

Start Recording | ON Light | ON Agitation | Backflush

DATA SOURCES

Live Data
44,084 events

1569 CRA2-2 — 5,696 events
12/15/2020 2:15:02 PM

1569-CRA2-1 — 10,000 events
12/15/2020 2:13:06 PM

1569-NC — 5,148 events
12/15/2020 2:10:54 PM

Update Compensation | Export FCS Files

Undo | Redo

Display Events 2,000 | Refresh Data

THRESHOLD AND SCATTER SETUP • Doublet Discrimination

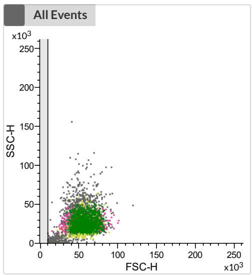

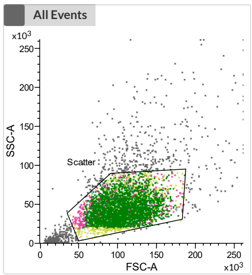

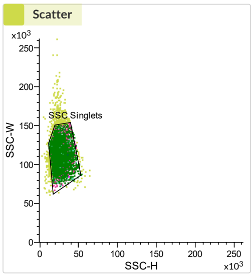

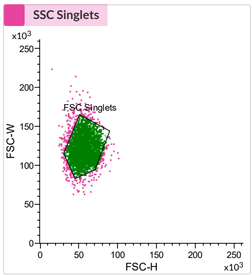

POPULATION HIERARCHY

- All Events
  - Scatter
    - SSC Singlets
      - FSC Singlets
        - PE pos
          - PE Sort
        - PE Neg

PLOTS

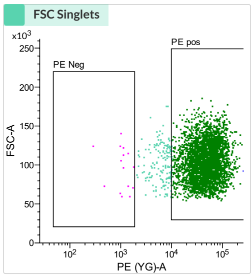

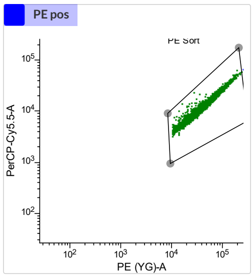

+

STATISTICS

Population	Events	% Parent	% Total	FSC-A Median	FSC-A %rCV	SSC-A Median	SSC-A %rCV
All Events	5,148		100.00 %	101612.30	31.47 %	41418.17	48.16 %
Scatter	4,348	84.46 %	84.46 %	102699.38	27.10 %	41033.62	39.62 %
SSC Singlets	3,865	88.89 %	75.08 %	103007.68	26.71 %	40820.95	36.26 %
FSC Singlets	3,529	91.31 %	68.55 %	103641.25	25.10 %	40464.64	35.29 %
PE pos	3,360	95.21 %	65.27 %	103834.35	24.97 %	40464.29	35.32 %
PE Sort	3,359	99.97 %	65.25 %	103883.77	24.97 %	40463.94	35.32 %
PE Neg	15	0.43 %	0.29 %	98194.36	37.78 %	33371.20	40.14 %

+

For Research Use Only. Not for use in diagnostic or therapeutic procedures.
