# Supplementary material for: Macropinocytosis mediates resistance to loss of glutamine transport in triple-negative breast cancer
Source: EMBO J. 2024 Oct 17;43(23):5857–82. doi: 10.1038/s44318-024-00271-6 (PMC11611898; doi:10.1038/s44318-024-00271-6)
Supplement: Supplementary file 5 — Source data Fig. 1 [file 44318_2024_271_MOESM5_ESM.zip › Figure 1/1J and K_FCS files/Sorting FCS files/20200225_231_NC, CRA2#1_ASCT2 sort/PE Pos Purity.pdf]

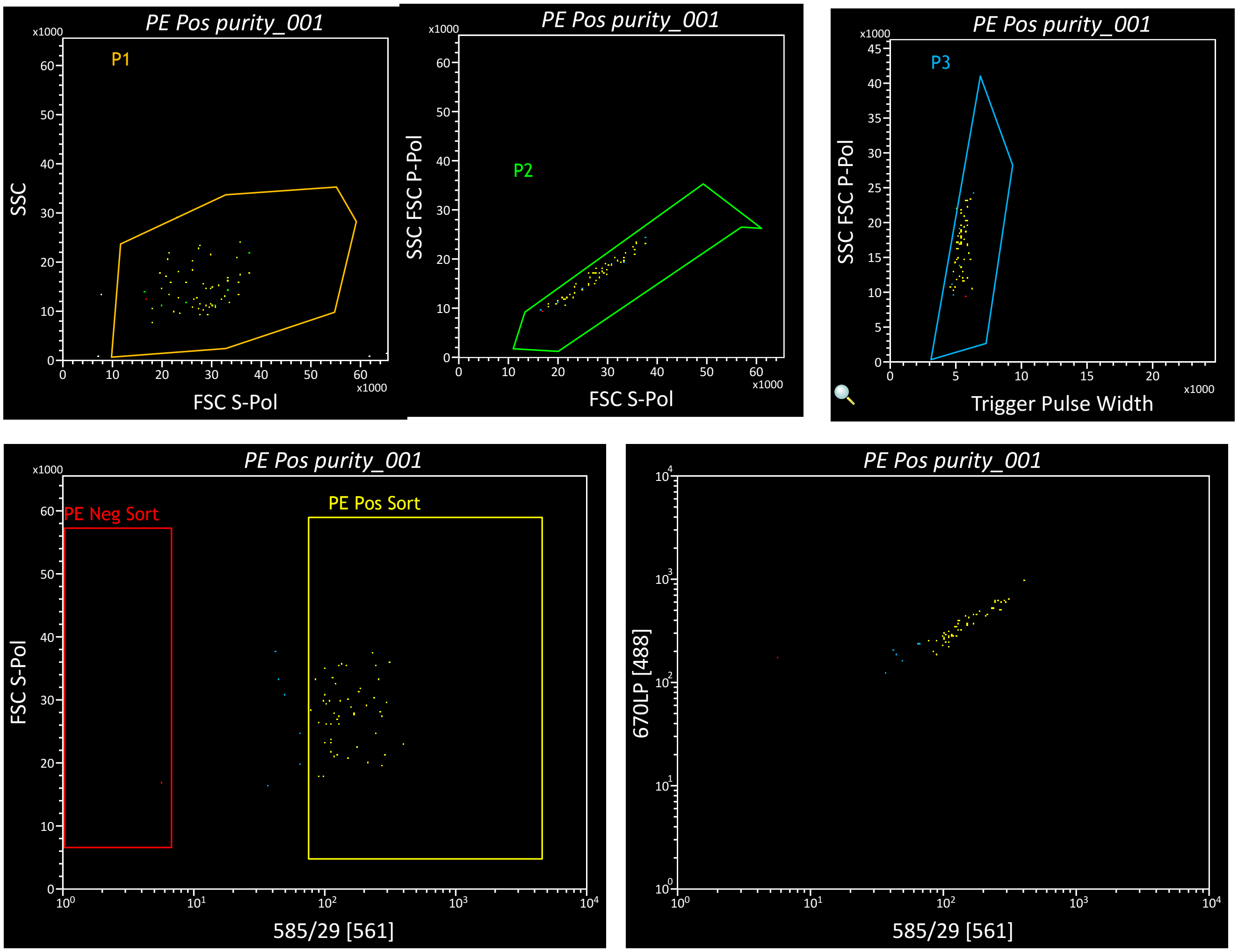

Populations: *PE Pos purity_001*

Populations	Events	% Total	% Parent
All Events	61	100.00%	####
P1	57	93.44%	93.44%
P2	57	93.44%	100.00%
P3	57	93.44%	100.00%
PE Neg Sort	1	1.64%	1.75%
PE Pos Sort	50	81.97%	87.72%
