# Supplementary material for: Macropinocytosis mediates resistance to loss of glutamine transport in triple-negative breast cancer
Source: EMBO J. 2024 Oct 17;43(23):5857–82. doi: 10.1038/s44318-024-00271-6 (PMC11611898; doi:10.1038/s44318-024-00271-6)
Supplement: Supplementary file 5 — Source data Fig. 1 [file 44318_2024_271_MOESM5_ESM.zip › Figure 1/1J and K_FCS files/Sorting FCS files/20200225_231_NC, CRA2#1_ASCT2 sort/Unstaiend.pdf]

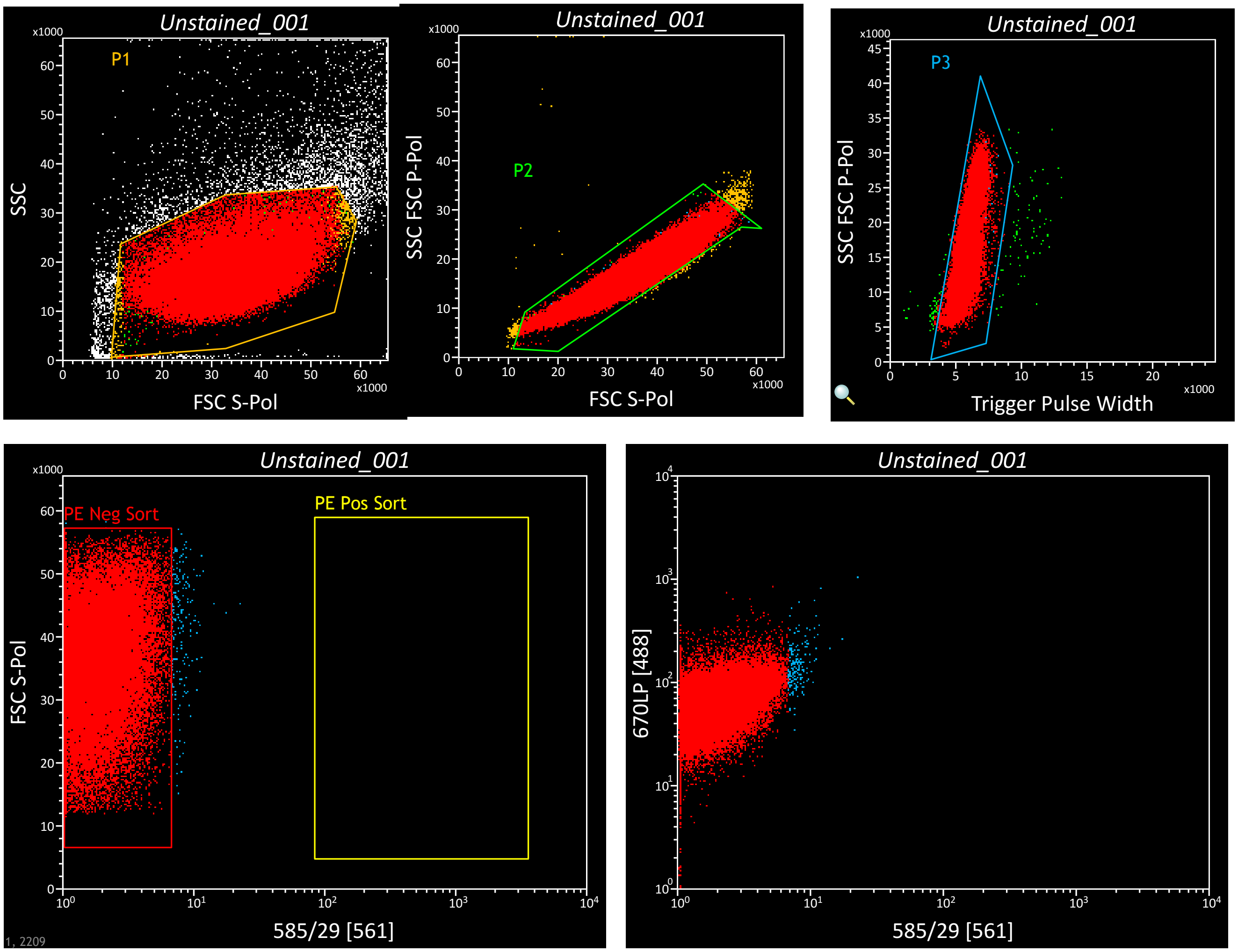

Populations: *Unstained_001*

Populations	Events	% Total	% Parent
All Events	54,750	100.00%	####
P1	51,207	93.53%	93.53%
P2	50,638	92.49%	98.89%
P3	50,512	92.26%	99.75%
PE Neg Sort	50,306	91.88%	99.59%
PE Pos Sort	0	0.00%	0.00%
