# Supplementary material for: Macropinocytosis mediates resistance to loss of glutamine transport in triple-negative breast cancer
Source: EMBO J. 2024 Oct 17;43(23):5857–82. doi: 10.1038/s44318-024-00271-6 (PMC11611898; doi:10.1038/s44318-024-00271-6)
Supplement: Supplementary file 5 — Source data Fig. 1 [file 44318_2024_271_MOESM5_ESM.zip › Figure 1/1J and K_FCS files/Sorting FCS files/20200225_231_NC, CRA2#1_ASCT2 sort/Sort report PE pos.pdf]

Sort Report

System

Sort Start:	2/25/2020 2:54:33 PM	Server:	Utopex
Application:	BD FACS™ Sortware	Build:	1.2.0.107
Version:	1.2.0.117	Cytometer Model:	BD Influx System
ValComp:	7.5.1.3.16	Cytometer Serial #:	X646500I2001

Details

Data Source:	Cytometer	Sort Mode:	1.0 Drop Pure
Nozzle Diameter (µm):	0.00	Drop Envelope:	1.0 Drop
Sheath Pressure (PSI):	0.00	Sort Objective:	Purify
Sort Device:	2 Tube Holder - 2 Way Sort	Phase Mask:	16/16
Piezo Amplitude:	5.85	Extra Coincidence Bits:	4
Drop Delay:	39.8736	Drop Frequency (kHz):	39.40

Sort Details

Name	Population	Event Limit	Event Count	Sort Count	Sort Rate	Aborts	Abort Rate	Efficiency	Time (sec)
Left	PE Pos Sort	Unlimited	1,111,741	399,318	415	31,773	33	92.6%	960
Right	-	-	-	-	-	-	-	-	-
