# Supplementary material for: Macropinocytosis mediates resistance to loss of glutamine transport in triple-negative breast cancer
Source: EMBO J. 2024 Oct 17;43(23):5857–82. doi: 10.1038/s44318-024-00271-6 (PMC11611898; doi:10.1038/s44318-024-00271-6)
Supplement: Supplementary file 5 — Source data Fig. 1 [file 44318_2024_271_MOESM5_ESM.zip › Figure 1/1J and K_FCS files/Sorting FCS files/20200807_MCF7_NC,CRA2#1_ASCT2 sort/US.pdf]

ACQUISITION DASHBOARD

Load Sample | Pause Sample | Flow Rate: 30 | Event Rate: 0 | Total Events: 0 | Processed Events: 0% | Elapsed Time: 00:36:01 | Recording Criteria: 10,000 | Population: All Events | 0 | 10,000 | Start Recording | ON Light | Agitation | Backflush

Undo | Redo | Display Events 2,000 | Refresh Data

DATA SOURCES

Live Data
0 events

CRA 2 — 10,000 events
08/07/2020 10:30:47 AM

NC — 10,000 events
08/07/2020 10:27:44 AM

US — 10,000 events
08/07/2020 10:24:35 AM

Update Compensation | Export FCS Files

POPULATION HIERARCHY

- All Events
  - Scatter
    - SSC Singlets
      - FSC Singlets
        - PE pos
          - PE Sort
        - PE Neg

THRESHOLD AND SCATTER SETUP • Doublet Discrimination

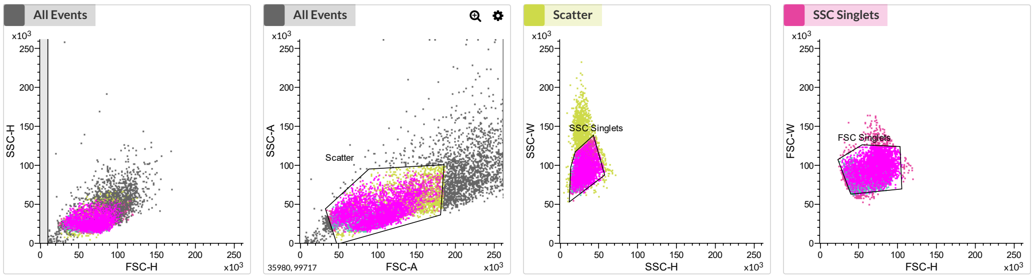

PLOTS

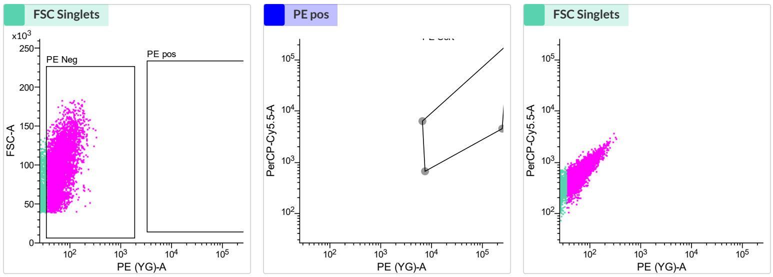

STATISTICS

Population	Events	% Parent	% Total	FSC-A Median	FSC-A %rCV	SSC-A Median	SSC-A %rCV
All Events	10,000		100.00 %	116660.25	57.59 %	45990.72	56.10 %
Scatter	7,216	72.16 %	72.16 %	99855.72	40.87 %	38375.37	39.03 %
SSC Singlets	6,112	84.70 %	61.12 %	93628.41	37.39 %	35969.40	34.61 %
FSC Singlets	5,799	94.88 %	57.99 %	92998.76	35.63 %	35549.59	33.43 %
PE pos	0	0.00 %	0.00 %				
PE Sort	0		0.00 %				
PE Neg	4,824	83.19 %	48.24 %	97897.64	32.44 %	37216.98	33.13 %
