# Supplementary material for: Macropinocytosis mediates resistance to loss of glutamine transport in triple-negative breast cancer
Source: EMBO J. 2024 Oct 17;43(23):5857–82. doi: 10.1038/s44318-024-00271-6 (PMC11611898; doi:10.1038/s44318-024-00271-6)
Supplement: Supplementary file 5 — Source data Fig. 1 [file 44318_2024_271_MOESM5_ESM.zip › Figure 1/1J and K_FCS files/Sorting FCS files/20200807_MCF7_NC,CRA2#1_ASCT2 sort/Sort Pos.pdf]

Sort Pos

CYTOMETER INFO

User Name:	Kanu Wahi	Application Name:	BD FACSChorus	Cytometer Serial Number:	R6627480006
Experiment Name:	KW20200807	Application Data Version:	1.1.19.0	Cytometer Name:	FACSMelody

SORT DETAILS

Sort Mode:	Purity	Sort Status:	Stopped by System	Start Date Time:	08/07/2020 11:04AM
Sort Device:	Tubes 5.0mL	Nozzle Size:	100 micron	End Date Time:	08/07/2020 11:39AM
Total Events:	829,208	Pressure:	22.90 PSI		
Processed Events:	100.0%	Drop Frequency:	34.0 kHz		

SORT STATISTICS

Tube	Population	Target Count	Sort Count	Sort Rate	Efficiency	Time
1	PE pos	1,008,000	484,239	229	98%	35m 7s

CYTOMETER SETTINGS

Fluorochrome	PMT Voltages
FSC	91
PE (YG)	455
SSC	397
GFP*	485
PerCP-Cy5.5	601

Compensation: Spillover Values

Into (Detectors)	From (Fluorochromes)		
	PE (YG)	GFP*	PerCP-Cy5.5
PE (YG)	100.00	0.00	0.01
GFP*	0.00	100.00	0.12
PerCP-Cy5.5	0.00	0.00	100.00

Threshold: FSC @ 10000

POPULATION HIERARCHY

- All Events
  - Scatter
    - SSC Singlets
      - FSC Singlets
        - PE pos
          - PE Sort
        - PE Neg
