# Supplementary figures and images for: Macropinocytosis mediates resistance to loss of glutamine transport in triple-negative breast cancer
Source: EMBO J. 2024 Oct 17;43(23):5857–82. doi: 10.1038/s44318-024-00271-6 (PMC11611898; doi:10.1038/s44318-024-00271-6)

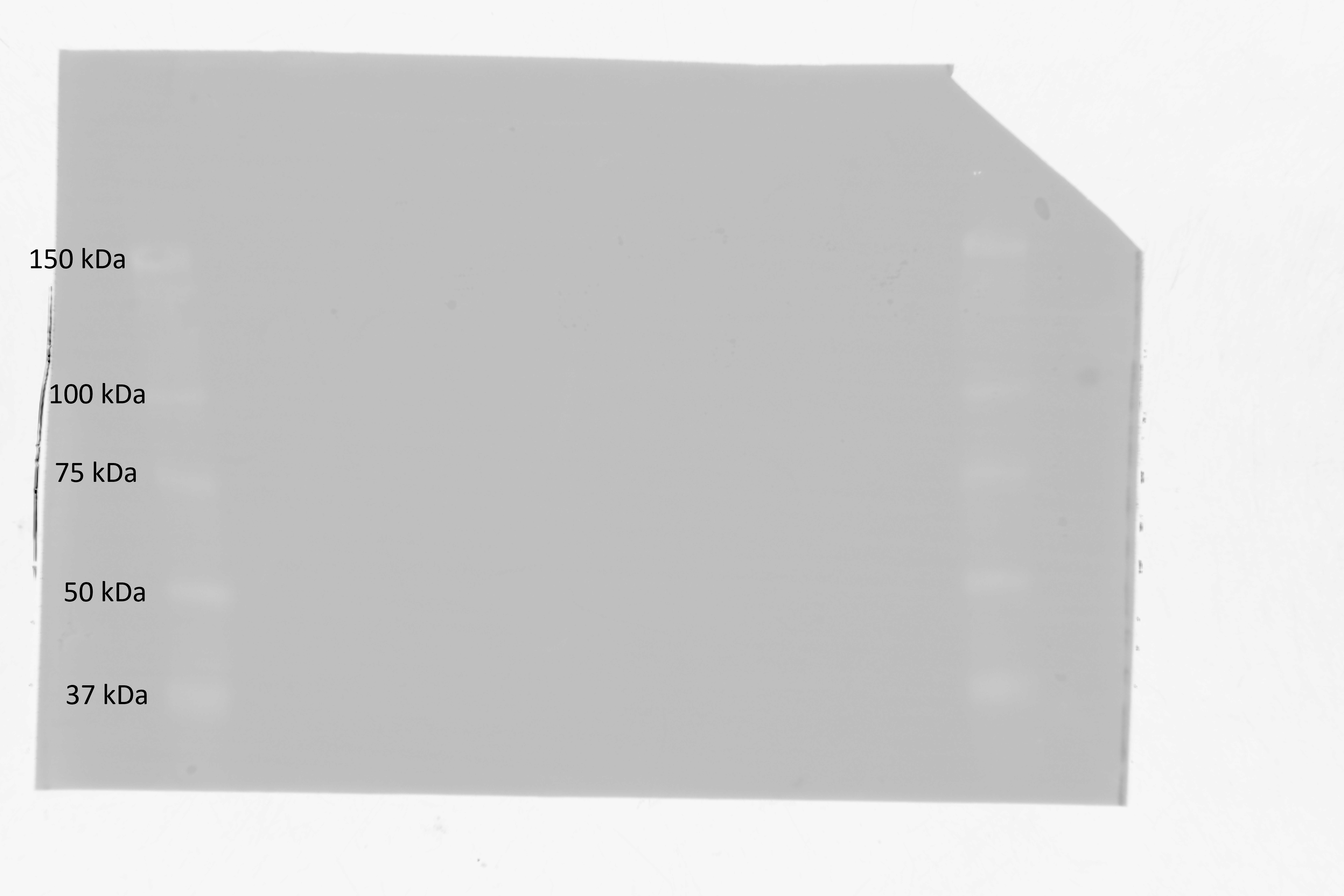

Supplement: Supplementary file 5 — Source data Fig. 1 [file 44318_2024_271_MOESM5_ESM.zip › Figure 1/1L and 1P/Labeled Marker_20200708_for ASCT2 and GAPDH.tif]

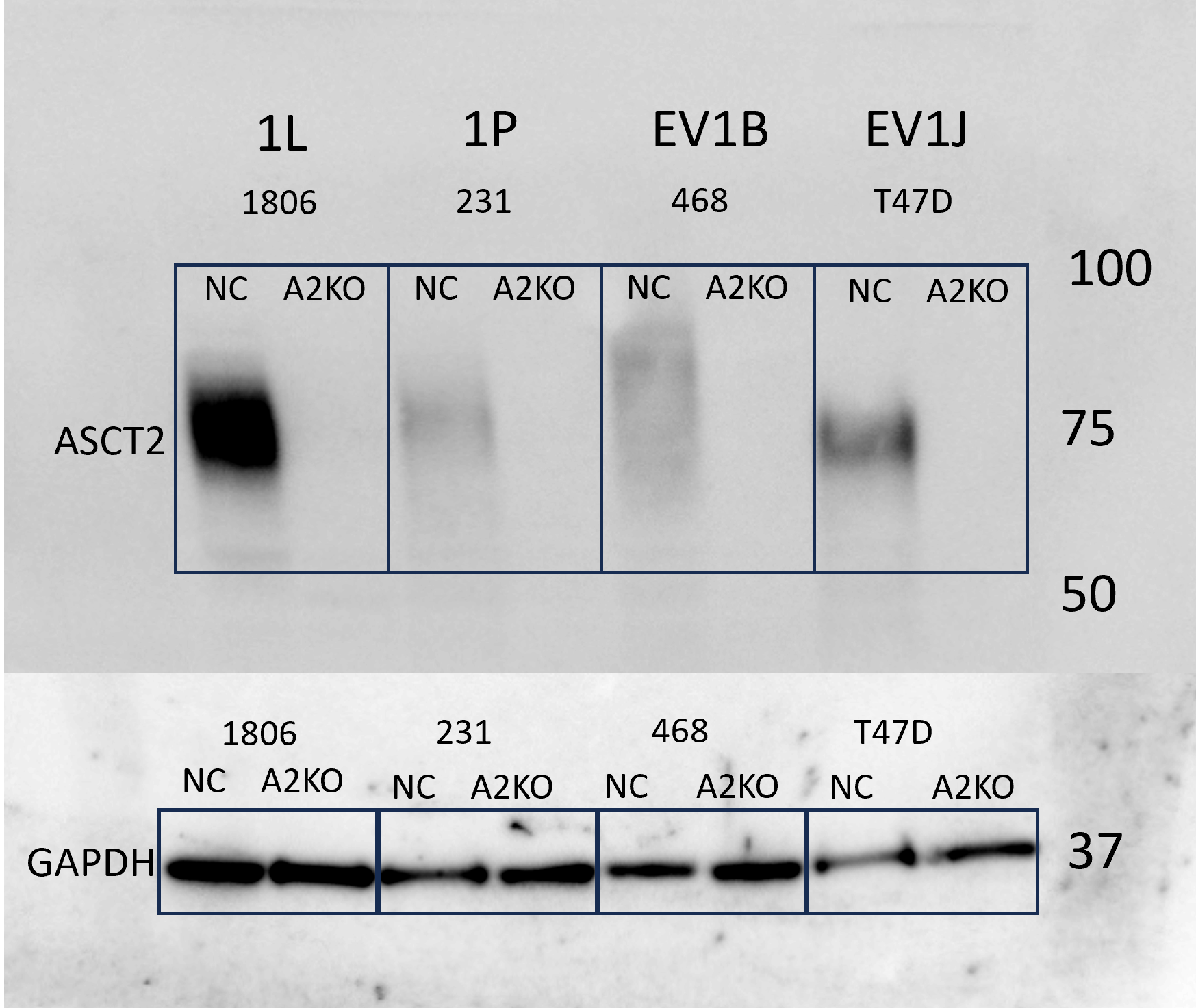

Supplement: Supplementary file 5 — Source data Fig. 1 [file 44318_2024_271_MOESM5_ESM.zip › Figure 1/1L and 1P/1L,P__20200708_ASCT2,GAPDH HCC WT KO.tif]

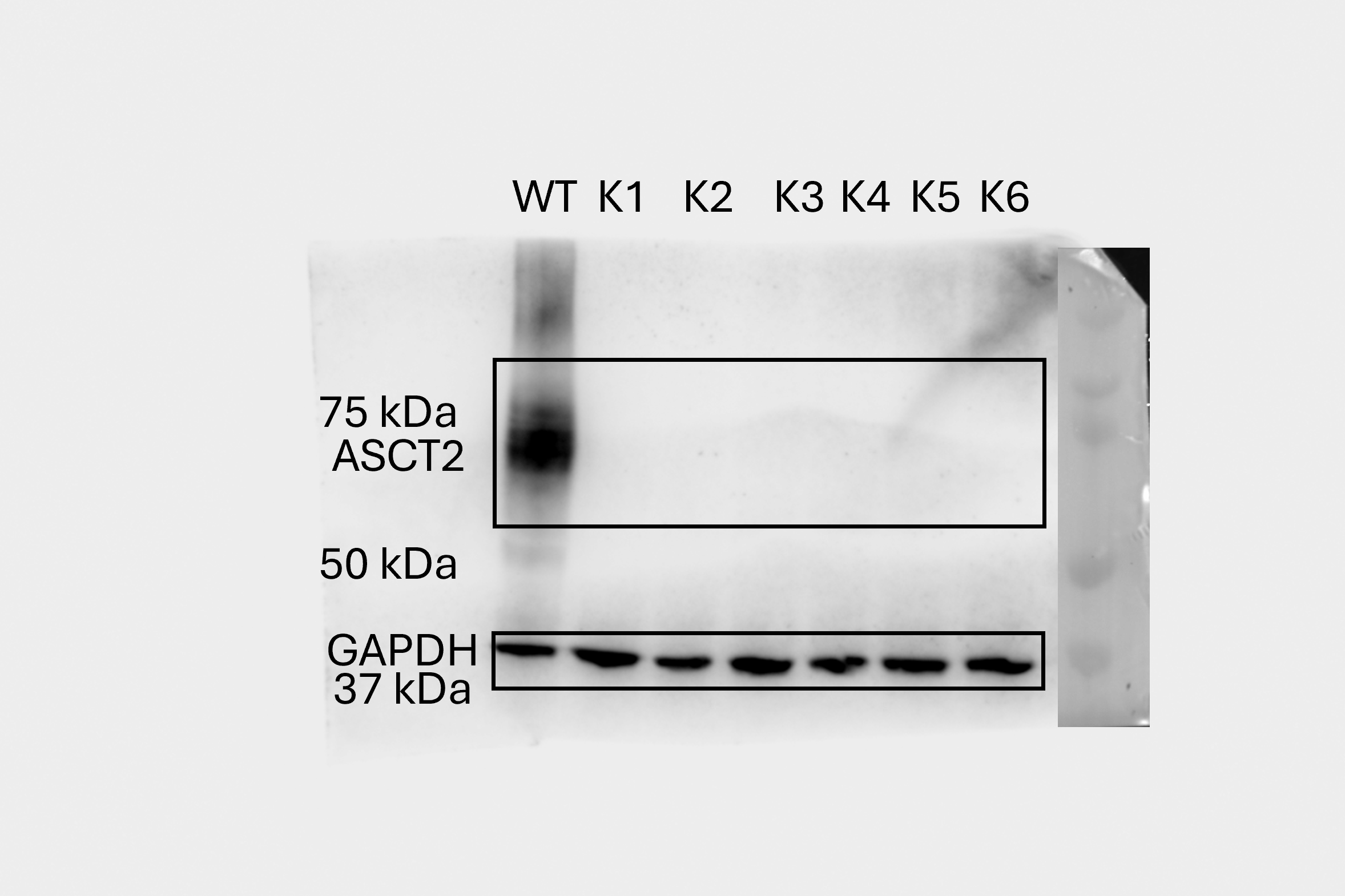

Supplement: Supplementary file 5 — Source data Fig. 1 [file 44318_2024_271_MOESM5_ESM.zip › Figure 1/1E/1E_20191108_ASCT2,GAPDH HCC WT KO_labels.tif]

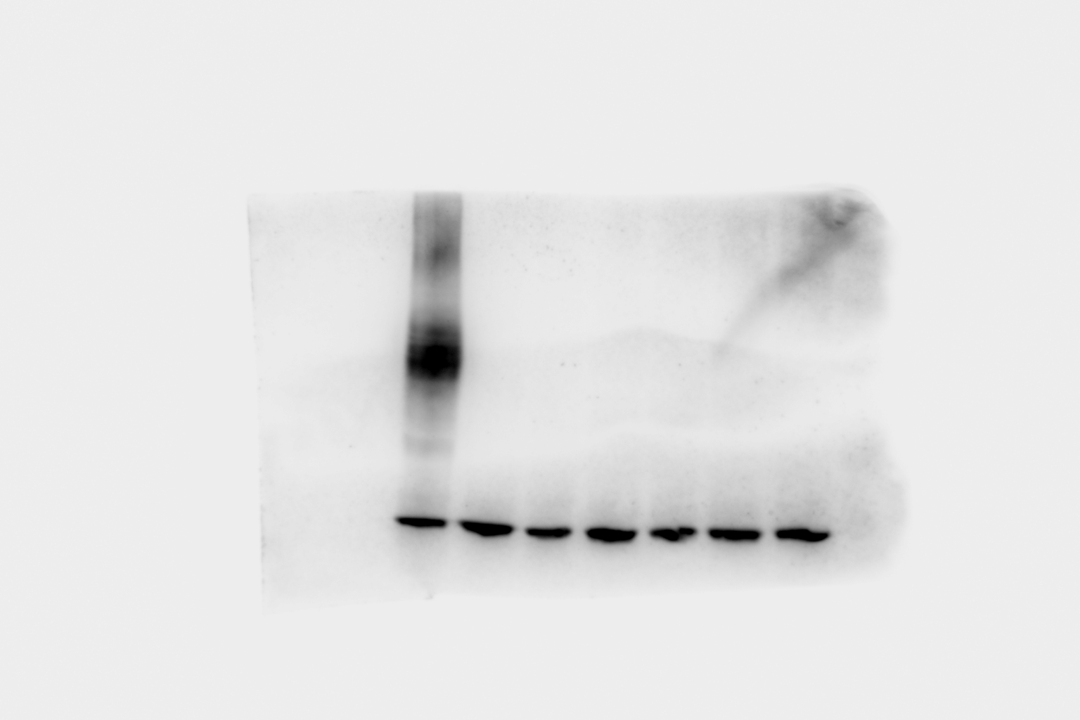

Supplement: Supplementary file 5 — Source data Fig. 1 [file 44318_2024_271_MOESM5_ESM.zip › Figure 1/1E/1E_20191108_ASCT2,GAPDH HCC WT KO.tif]

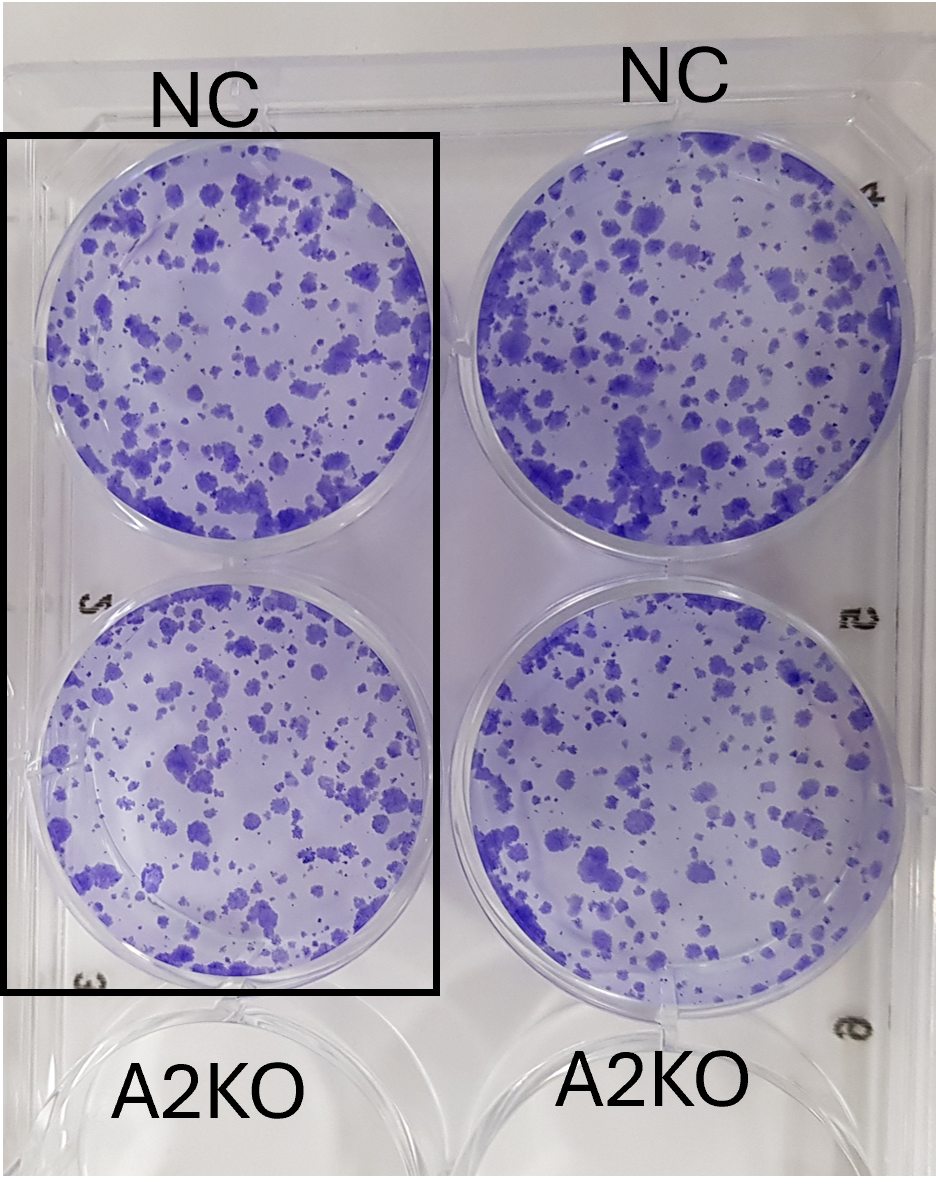

Supplement: Supplementary file 5 — Source data Fig. 1 [file 44318_2024_271_MOESM5_ESM.zip › Figure 1/1O and 1S/1O and 1S/1O_HCC1806_Colony formation assay.tif]

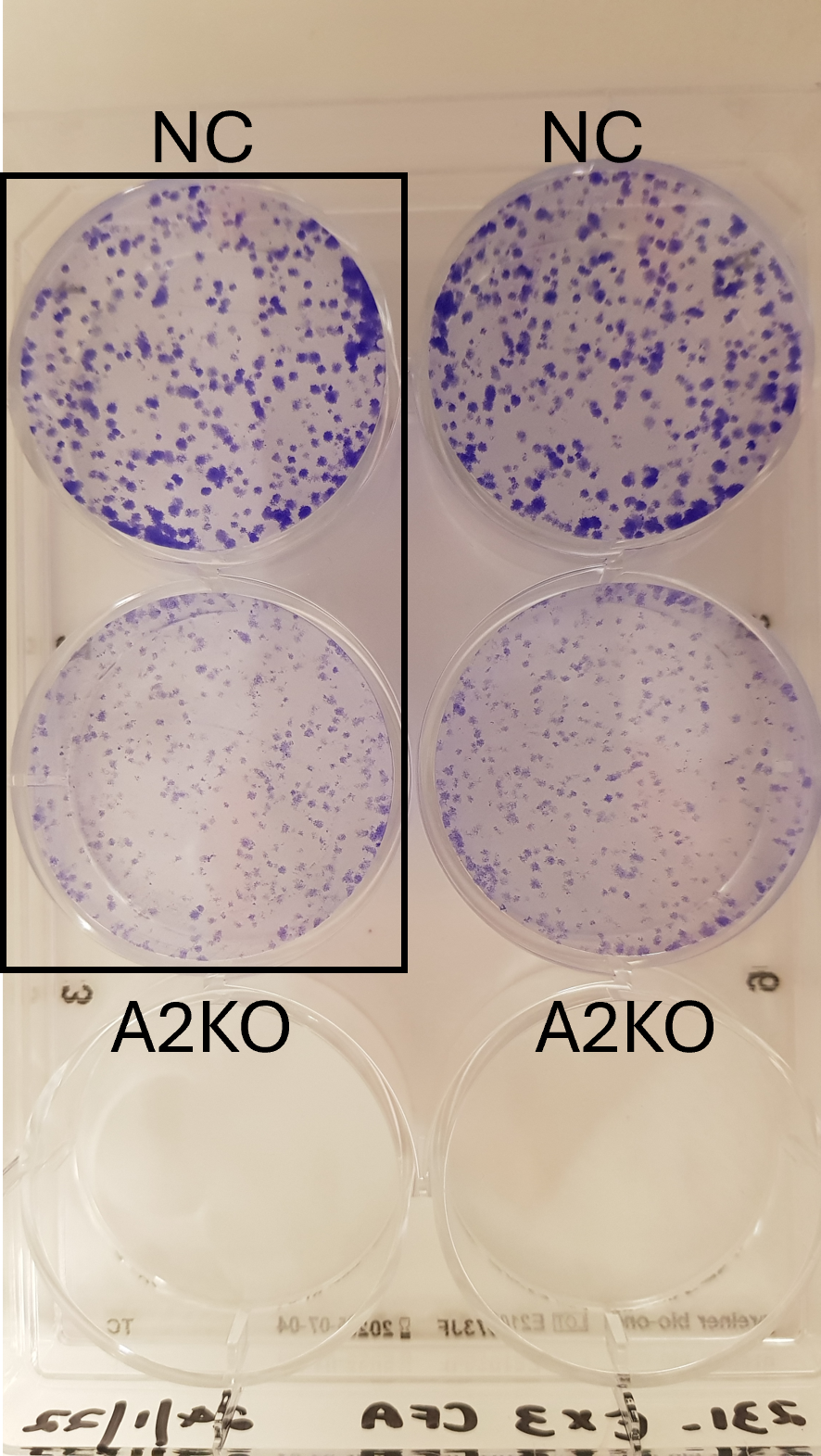

Supplement: Supplementary file 5 — Source data Fig. 1 [file 44318_2024_271_MOESM5_ESM.zip › Figure 1/1O and 1S/1O and 1S/1S_MDA-MB-231_Colony formation assay.tif]

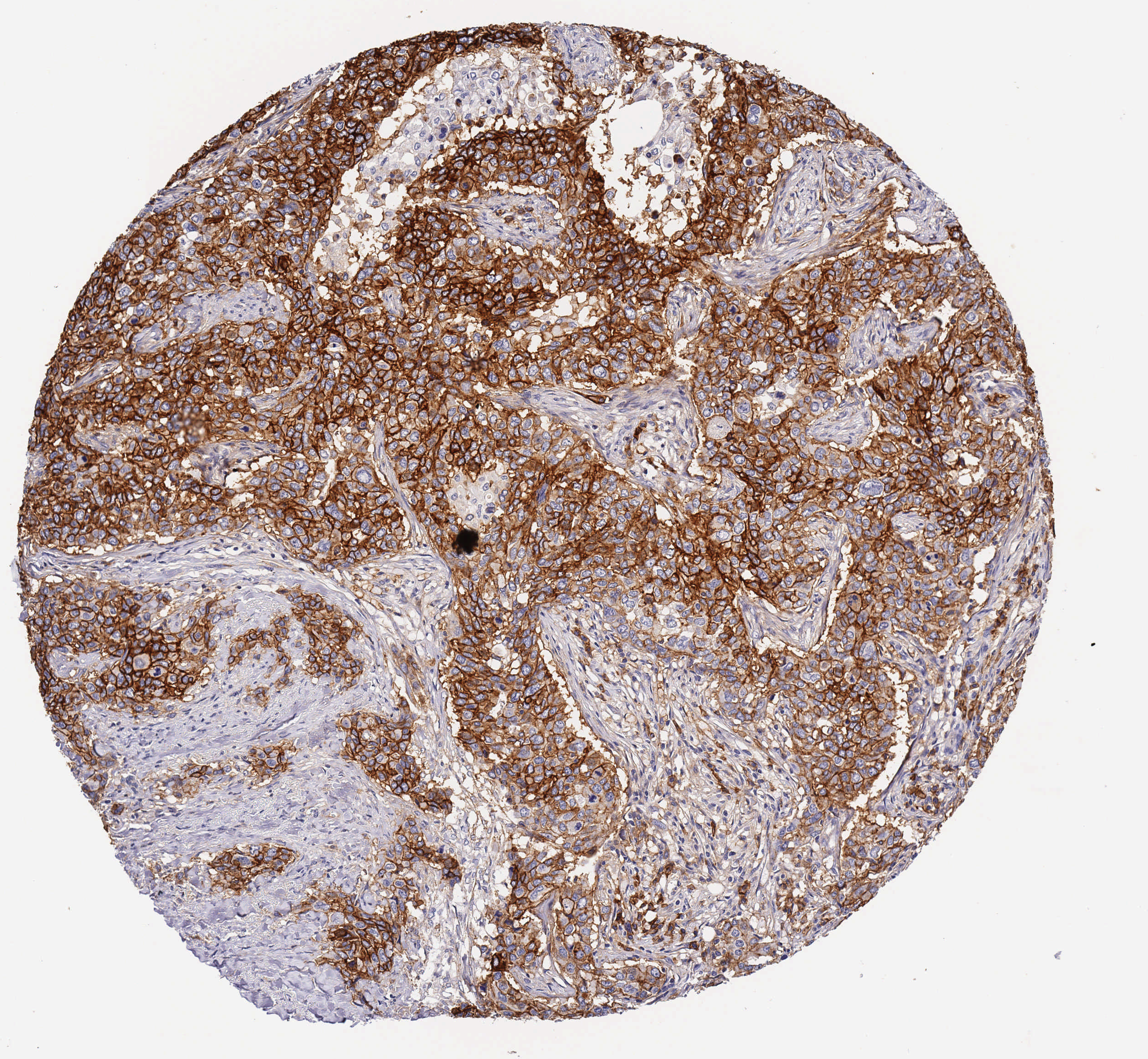

Supplement: Supplementary file 5 — Source data Fig. 1 [file 44318_2024_271_MOESM5_ESM.zip › Figure 1/1A, B and C/1A/RPAH TNBC TMA#2 ASCT2 Exp2 D6.tif]

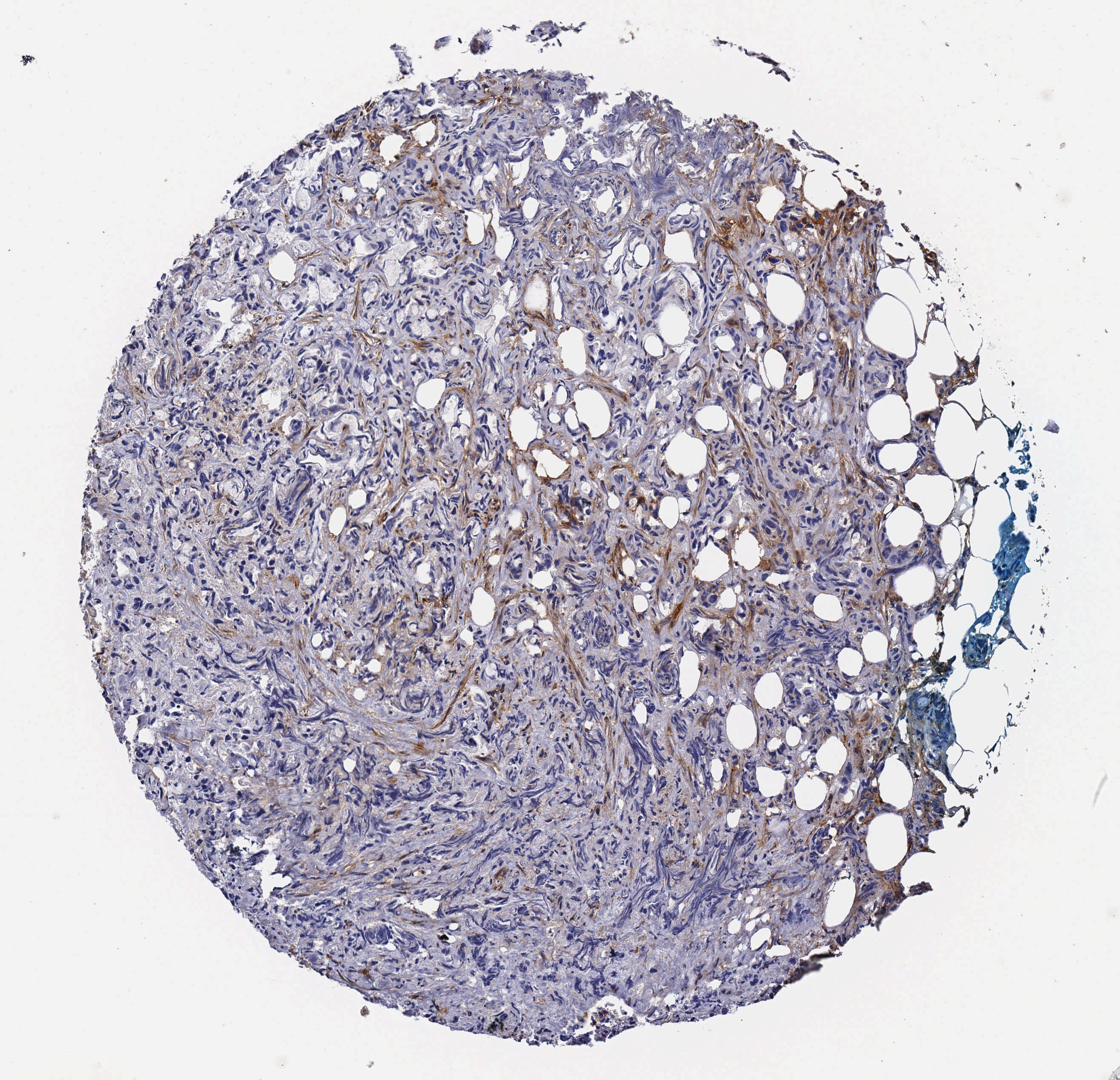

Supplement: Supplementary file 5 — Source data Fig. 1 [file 44318_2024_271_MOESM5_ESM.zip › Figure 1/1A, B and C/1A/RPAH TNBC TMA#1 ASCT2 Exp0 B1.tif]

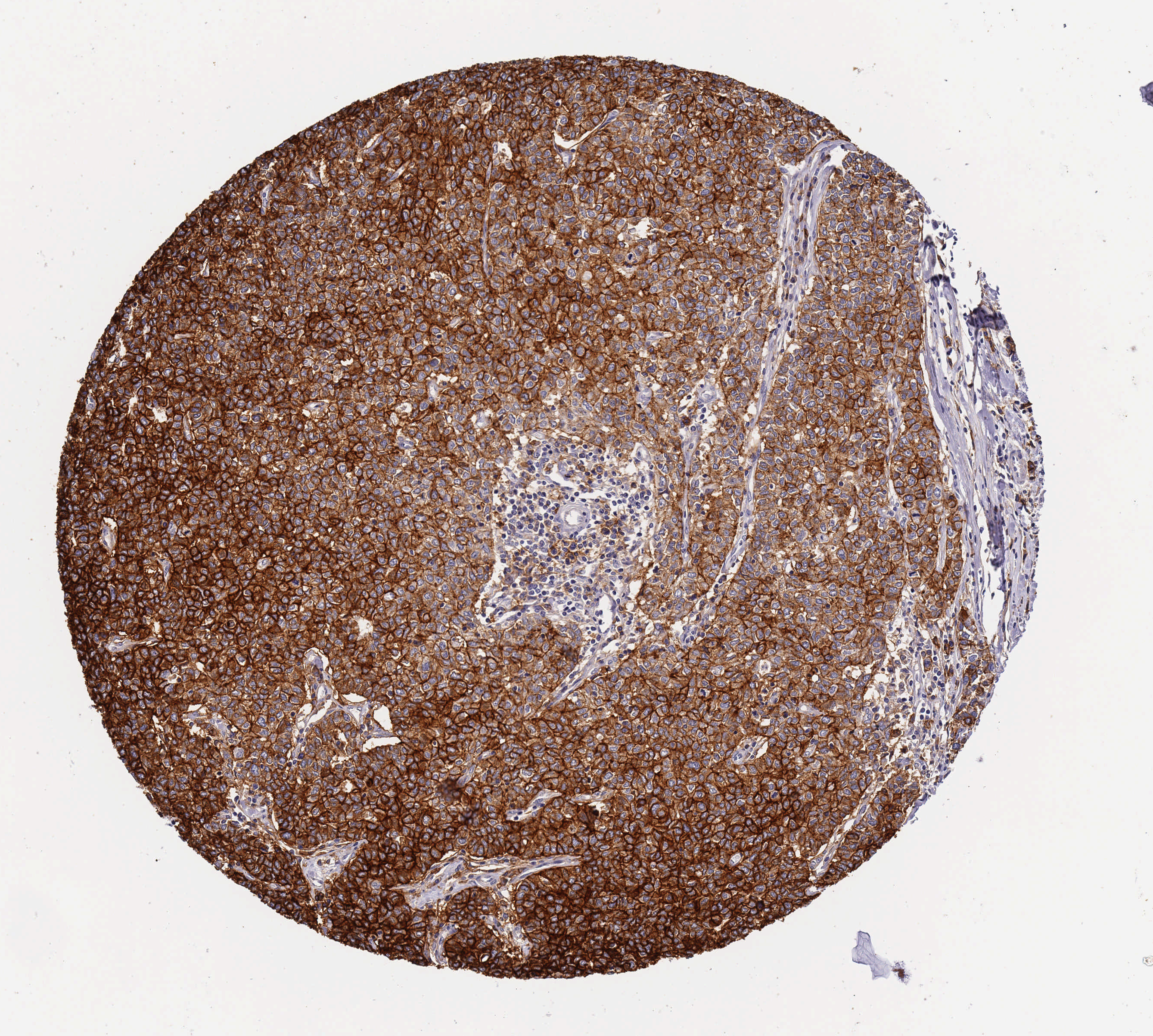

Supplement: Supplementary file 5 — Source data Fig. 1 [file 44318_2024_271_MOESM5_ESM.zip › Figure 1/1A, B and C/1A/RPAH TNBC TMA#2 ASCT2 Exp3 H5.tif]

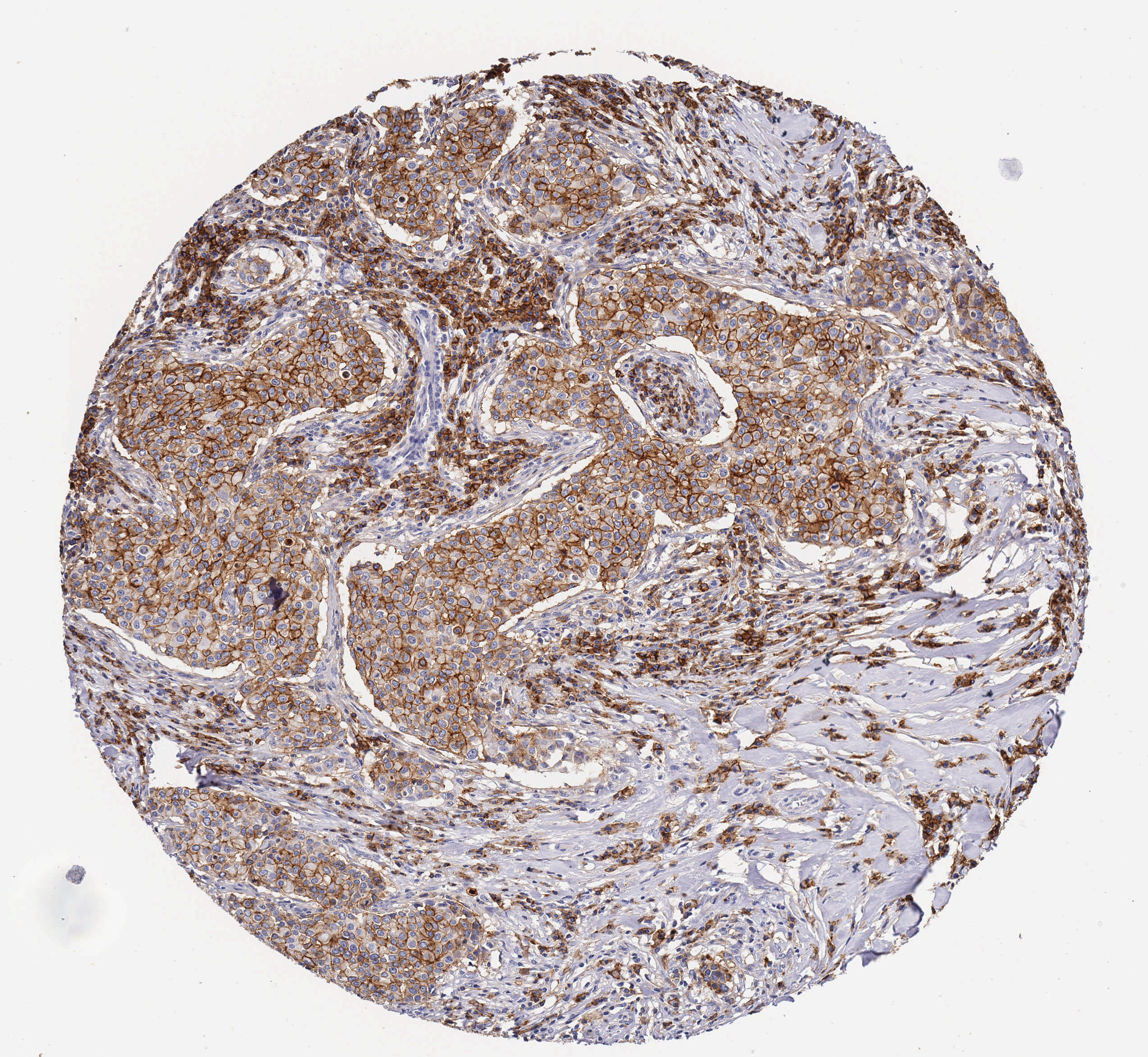

Supplement: Supplementary file 5 — Source data Fig. 1 [file 44318_2024_271_MOESM5_ESM.zip › Figure 1/1A, B and C/1A/RPAH TNBC TMA#2 ASCT2 Exp1 D1.tif]

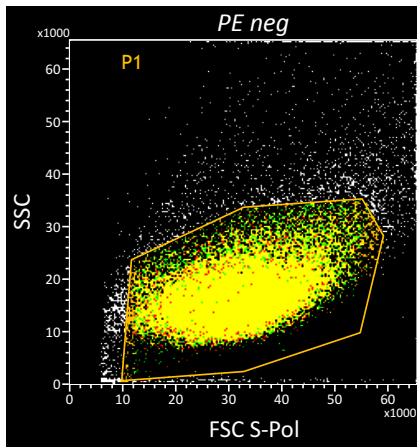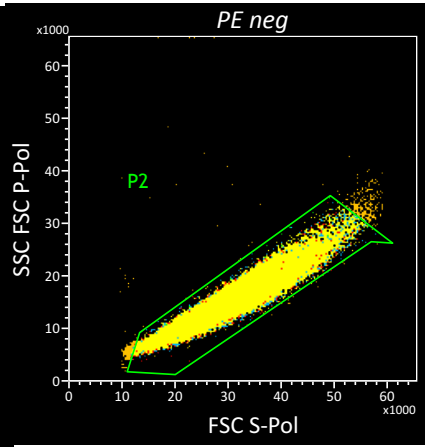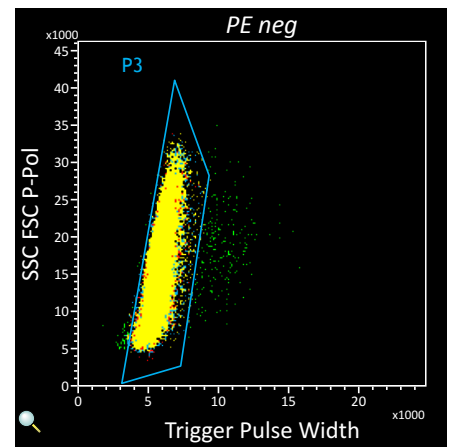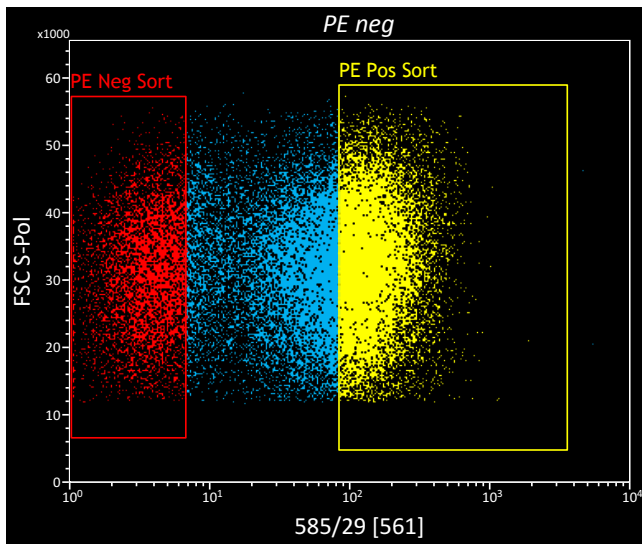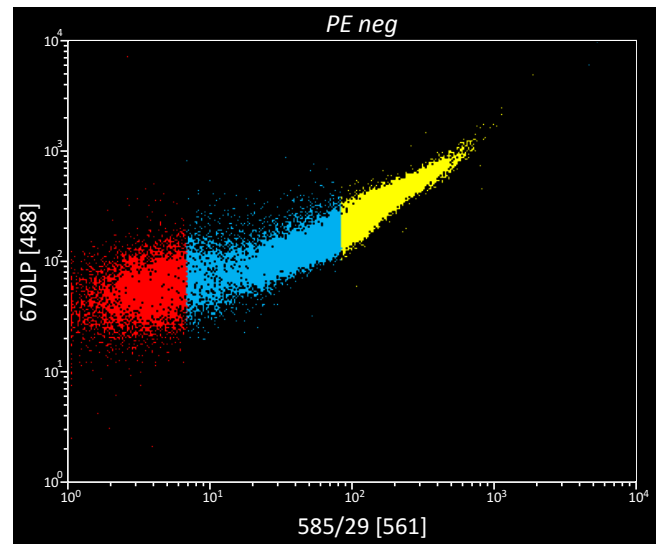

Populations: *PE neg*

| Populations | Events | % Total | % Parent |
|-------------|--------|---------|----------|
| All Events  | 38,458 | 100.00% | ####     |
| P1          | 36,074 | 93.80%  | 93.80%   |
| P2          | 35,529 | 92.38%  | 98.49%   |
| P3          | 35,275 | 91.72%  | 99.29%   |
| PE Neg Sort | 6,053  | 15.74%  | 17.16%   |
| PE Pos Sort | 16,211 | 42.15%  | 45.96%   |

Supplement: Supplementary file 5 — Source data Fig. 1 [file 44318_2024_271_MOESM5_ESM.zip › Figure 1/1J and K_FCS files/Sorting FCS files/20200225_231_NC, CRA2#1_ASCT2 sort/PE neg.pdf]

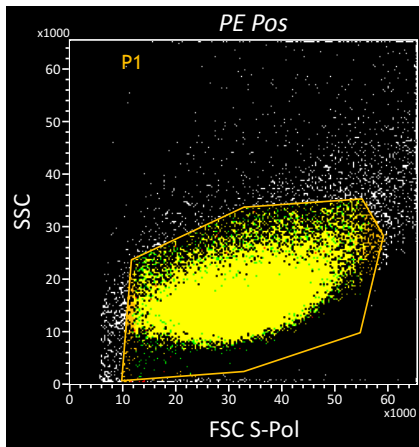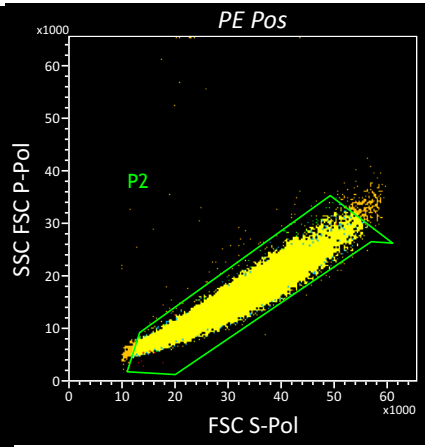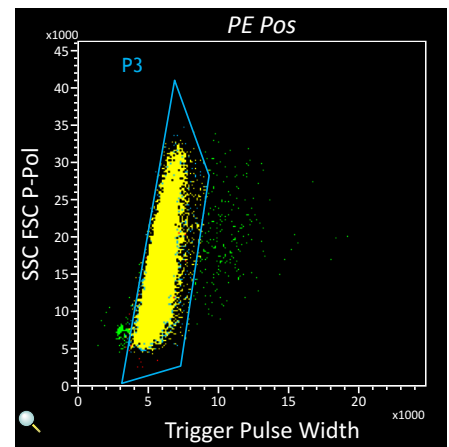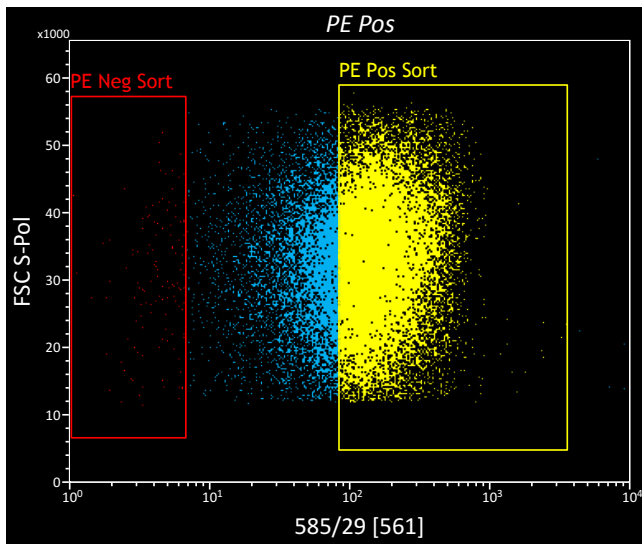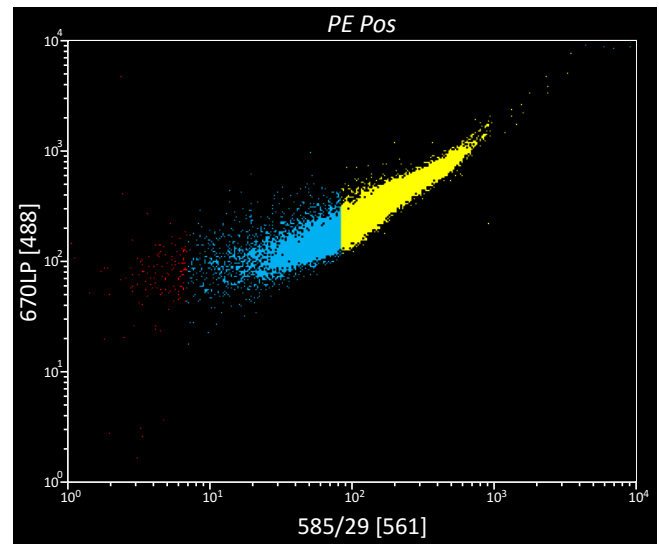

| Populations: PE Pos |        |         |          |
|---------------------|--------|---------|----------|
| Populations         | Events | % Total | % Parent |
| All Events          | 35,321 | 100.00% | ####     |
| P1                  | 33,271 | 94.20%  | 94.20%   |
| P2                  | 32,677 | 92.51%  | 98.21%   |
| P3                  | 32,360 | 91.62%  | 99.03%   |
| PE Neg Sort         | 135    | 0.38%   | 0.42%    |
| PE Pos Sort         | 24,769 | 70.13%  | 76.54%   |

Supplement: Supplementary file 5 — Source data Fig. 1 [file 44318_2024_271_MOESM5_ESM.zip › Figure 1/1J and K_FCS files/Sorting FCS files/20200225_231_NC, CRA2#1_ASCT2 sort/PE Pos.pdf]

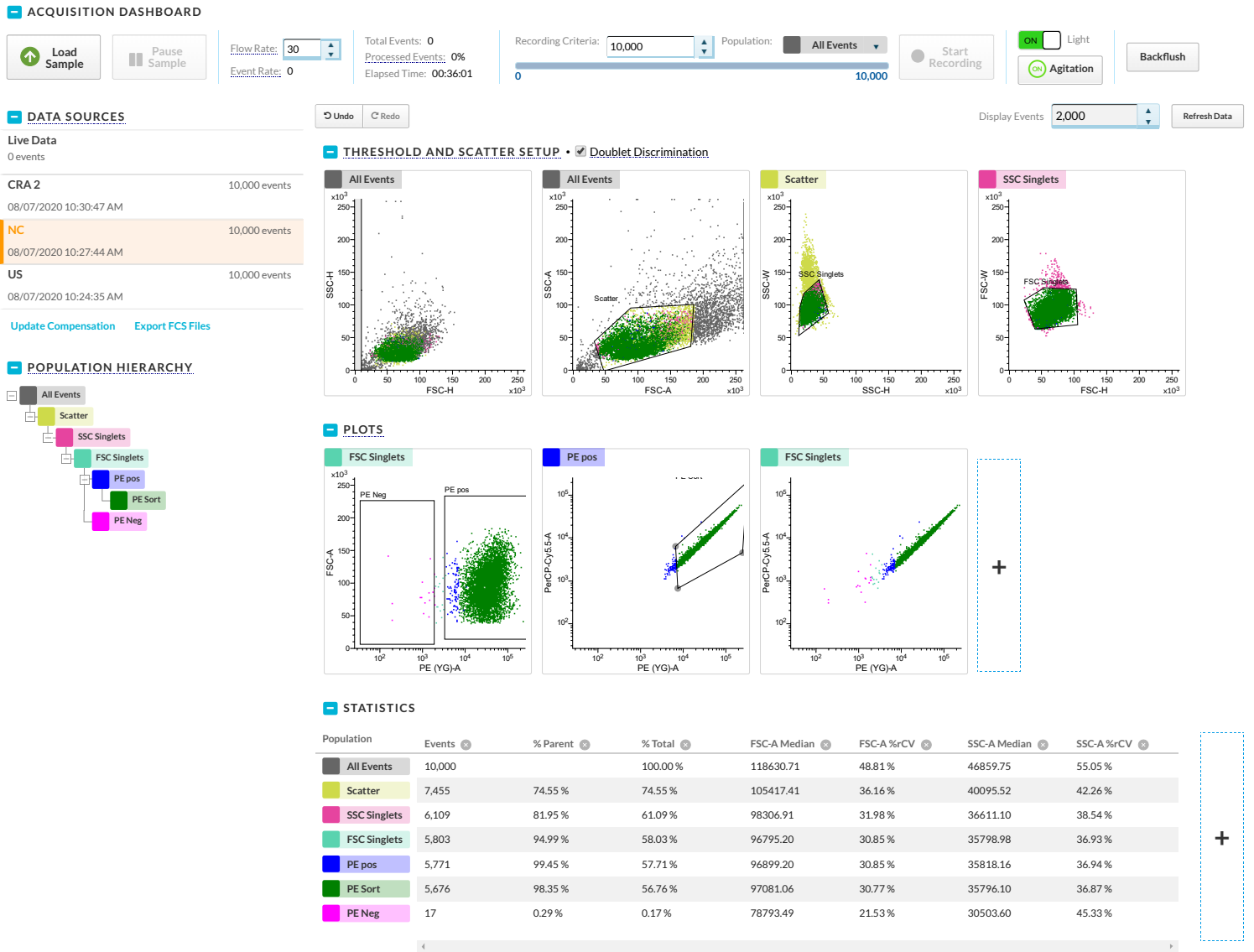

Supplement: Supplementary file 5 — Source data Fig. 1 [file 44318_2024_271_MOESM5_ESM.zip › Figure 1/1J and K_FCS files/Sorting FCS files/20200807_MCF7_NC,CRA2#1_ASCT2 sort/NC.pdf]

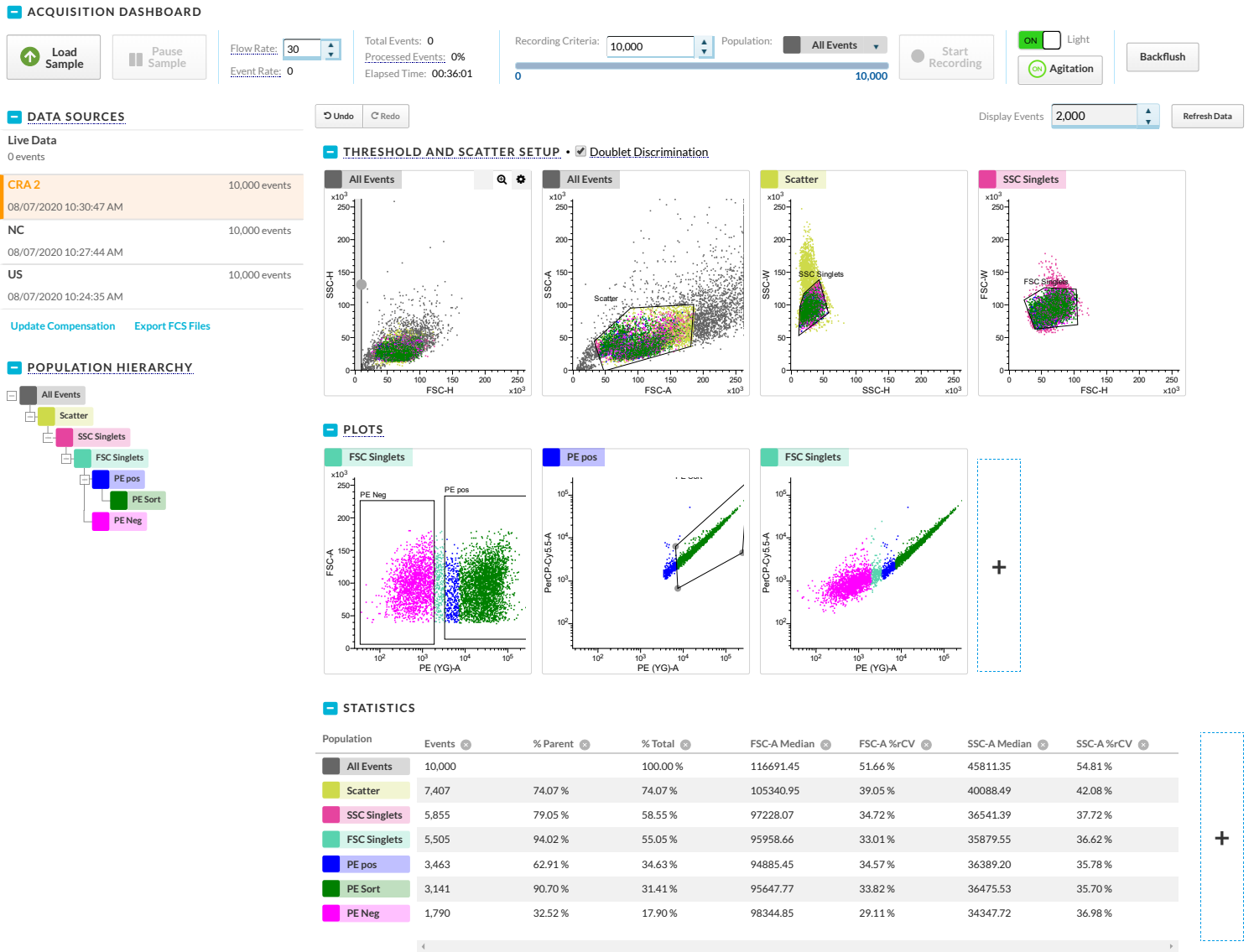

Supplement: Supplementary file 5 — Source data Fig. 1 [file 44318_2024_271_MOESM5_ESM.zip › Figure 1/1J and K_FCS files/Sorting FCS files/20200807_MCF7_NC,CRA2#1_ASCT2 sort/CRA 2.pdf]

# BD FACSDiva 8.0

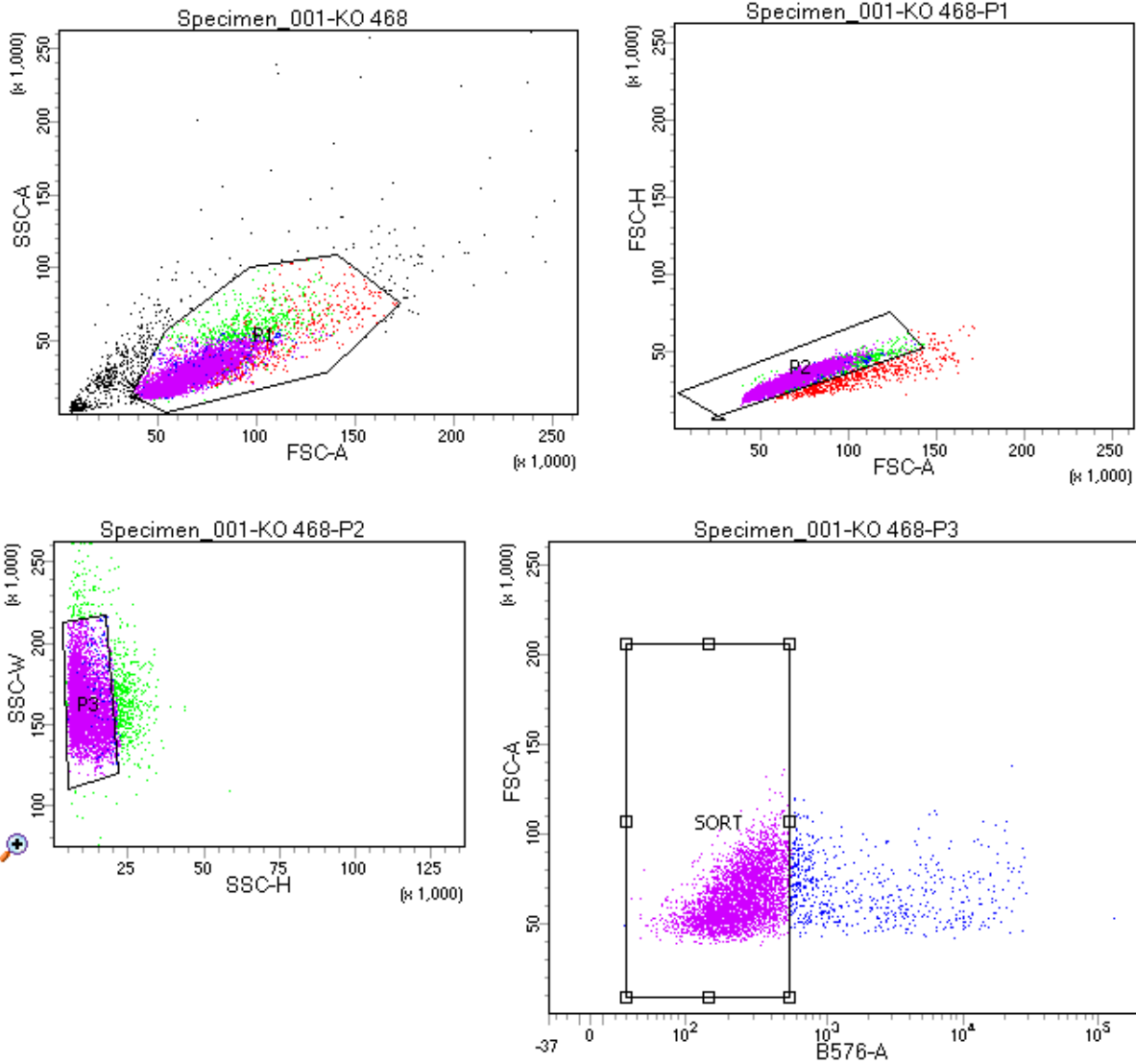

| Tube: KO 468 |         |         |        |
|--------------|---------|---------|--------|
| Population   | #Events | %Parent | %Total |
| ■ All Events | 6,510   | ####    | 100.0  |
| ■ P1         | 5,853   | 89.9    | 89.9   |
| ■ P2         | 5,208   | 89.0    | 80.0   |
| ■ P3         | 4,575   | 87.8    | 70.3   |
| ■ SORT       | 3,969   | 86.8    | 61.0   |

Supplement: Supplementary file 5 — Source data Fig. 1 [file 44318_2024_271_MOESM5_ESM.zip › Figure 1/1J and K_FCS files/Sorting FCS files/20200310_468_NC, CRA2#1_ASCT2 sort/KO 468.pdf]

# BD FACSDiva 8.0

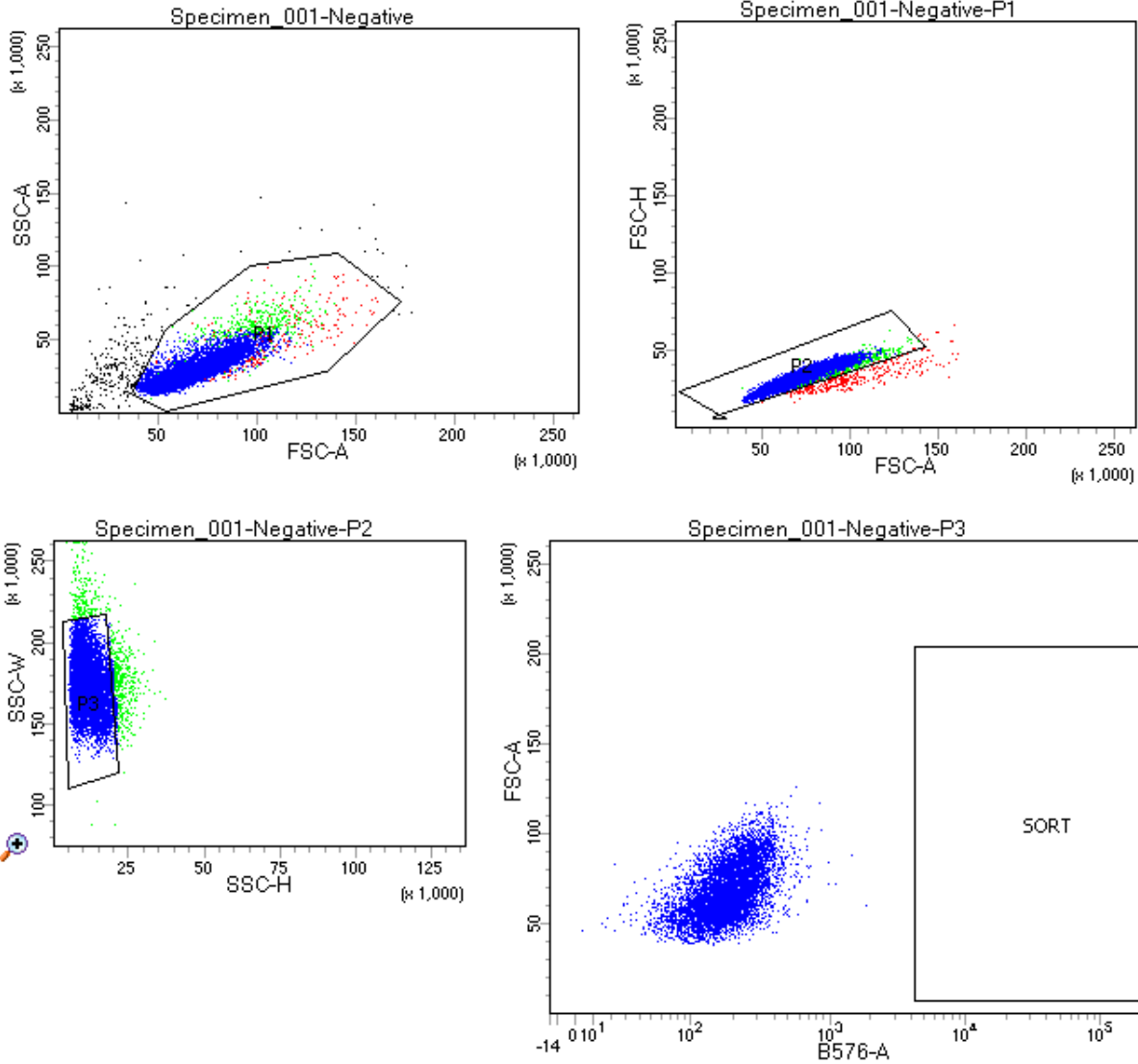

| Tube: Negative |         |         |        |
|----------------|---------|---------|--------|
| Population     | #Events | %Parent | %Total |
| ■ All Events   | 8,026   | ####    | 100.0  |
| ■ P1           | 7,670   | 95.6    | 95.6   |
| ■ P2           | 7,328   | 95.5    | 91.3   |
| ■ P3           | 6,740   | 92.0    | 84.0   |
| ■ SORT         | 0       | 0.0     | 0.0    |

Supplement: Supplementary file 5 — Source data Fig. 1 [file 44318_2024_271_MOESM5_ESM.zip › Figure 1/1J and K_FCS files/Sorting FCS files/20200310_468_NC, CRA2#1_ASCT2 sort/Negative.pdf]

# BD FACSDiva 8.0

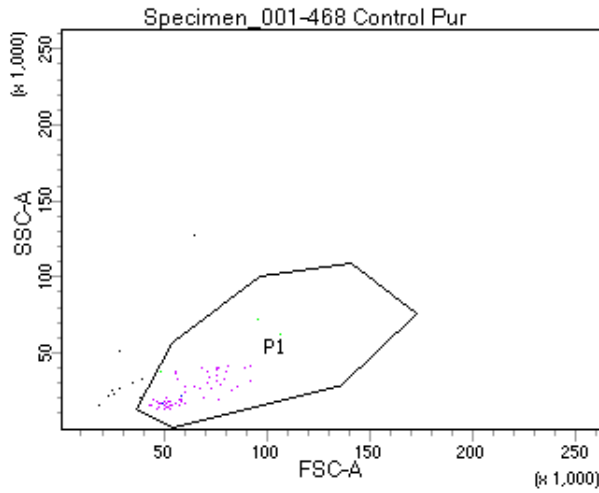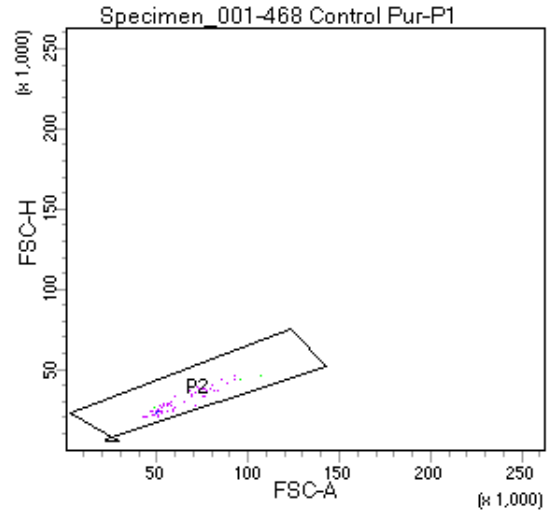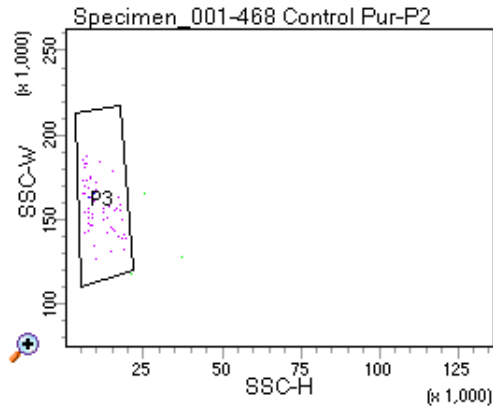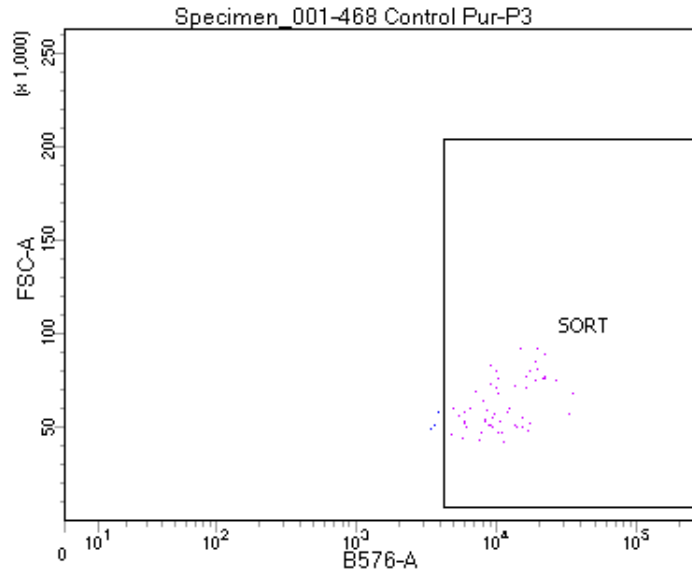

| Tube: 468 Control Pur |         |         |        |
|-----------------------|---------|---------|--------|
| Population            | #Events | %Parent | %Total |
| ■ All Events          | 71      | ####    | 100.0  |
| ■ P1                  | 61      | 85.9    | 85.9   |
| ■ P2                  | 61      | 100.0   | 85.9   |
| ■ P3                  | 58      | 95.1    | 81.7   |
| ■ SORT                | 55      | 94.8    | 77.5   |

Supplement: Supplementary file 5 — Source data Fig. 1 [file 44318_2024_271_MOESM5_ESM.zip › Figure 1/1J and K_FCS files/Sorting FCS files/20200310_468_NC, CRA2#1_ASCT2 sort/468 Control Pur.pdf]

# BD FACSDiva 8.0

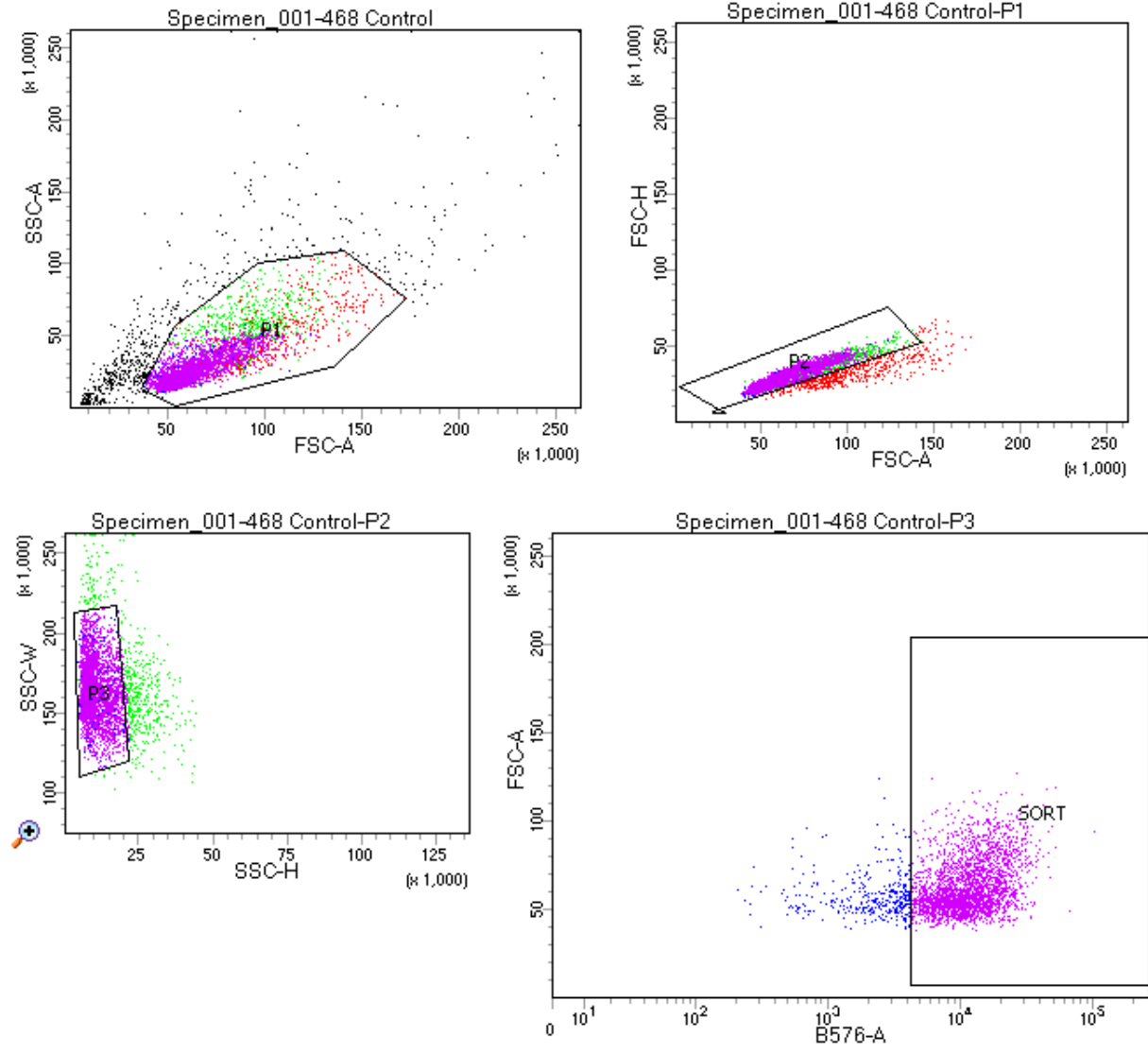

| Tube: 468 Control |         |         |        |
|-------------------|---------|---------|--------|
| Population        | #Events | %Parent | %Total |
| ■ All Events      | 4,898   | ####    | 100.0  |
| ■ P1              | 4,278   | 87.3    | 87.3   |
| ■ P2              | 3,675   | 85.9    | 75.0   |
| ■ P3              | 3,124   | 85.0    | 63.8   |
| ■ SORT            | 2,749   | 88.0    | 56.1   |

Supplement: Supplementary file 5 — Source data Fig. 1 [file 44318_2024_271_MOESM5_ESM.zip › Figure 1/1J and K_FCS files/Sorting FCS files/20200310_468_NC, CRA2#1_ASCT2 sort/468 Control.pdf]

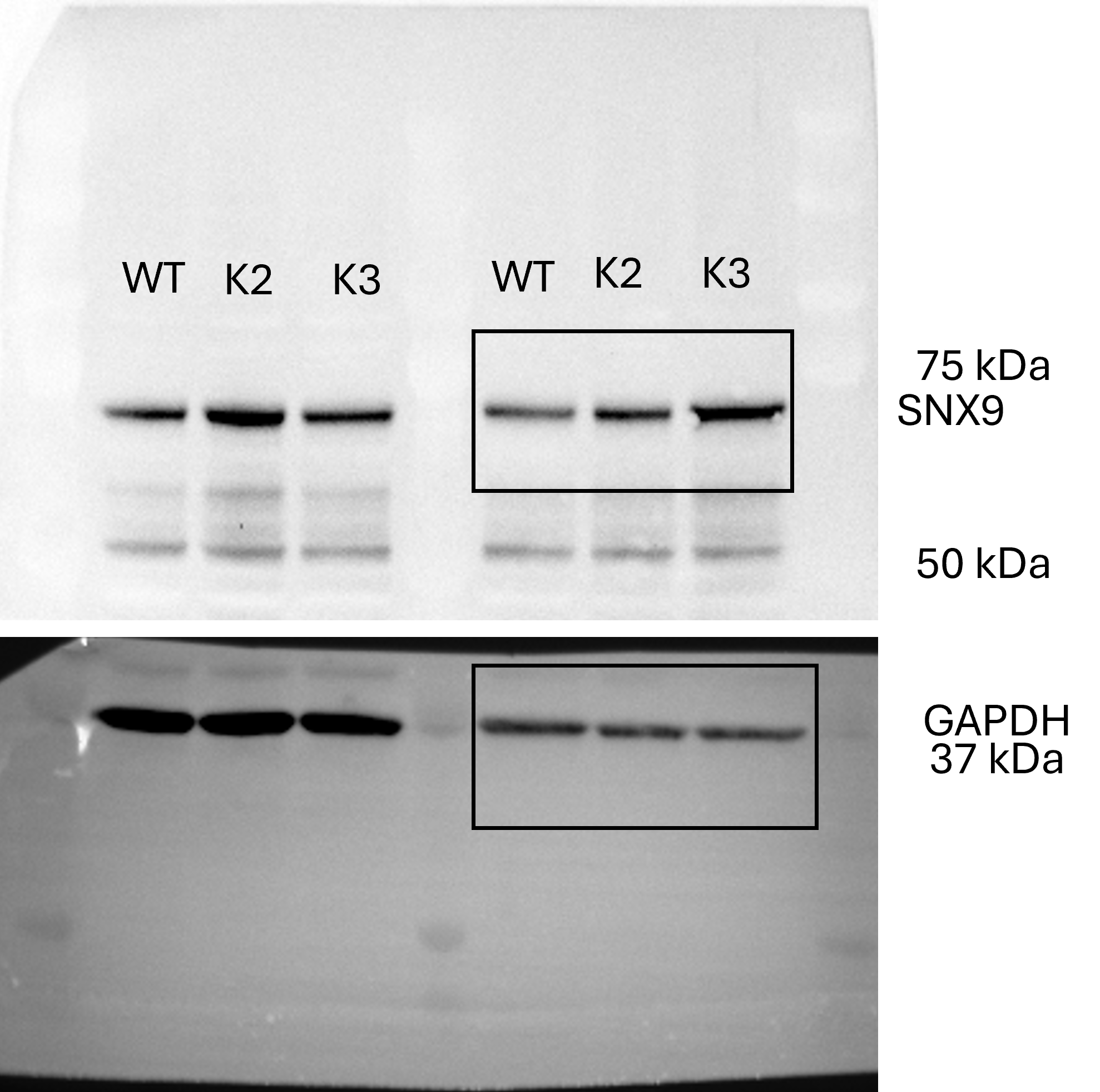

Supplement: Supplementary file 8 — Source data Fig. 4 [file 44318_2024_271_MOESM8_ESM.zip › Figure 4/4D_SNX9 WB.tif]

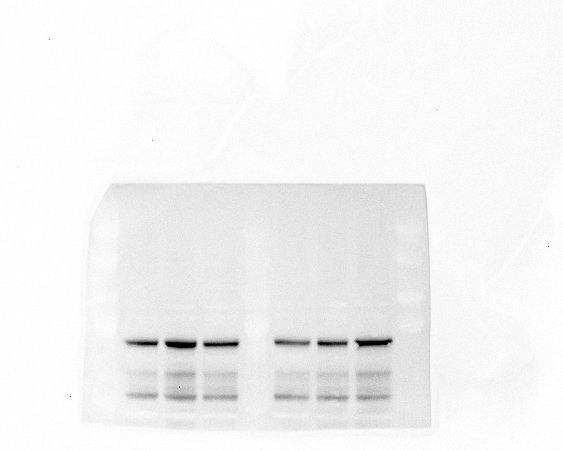

Supplement: Supplementary file 8 — Source data Fig. 4 [file 44318_2024_271_MOESM8_ESM.zip › Figure 4/06202024_SNX9_1_1806_WT_K2_K3.tif]

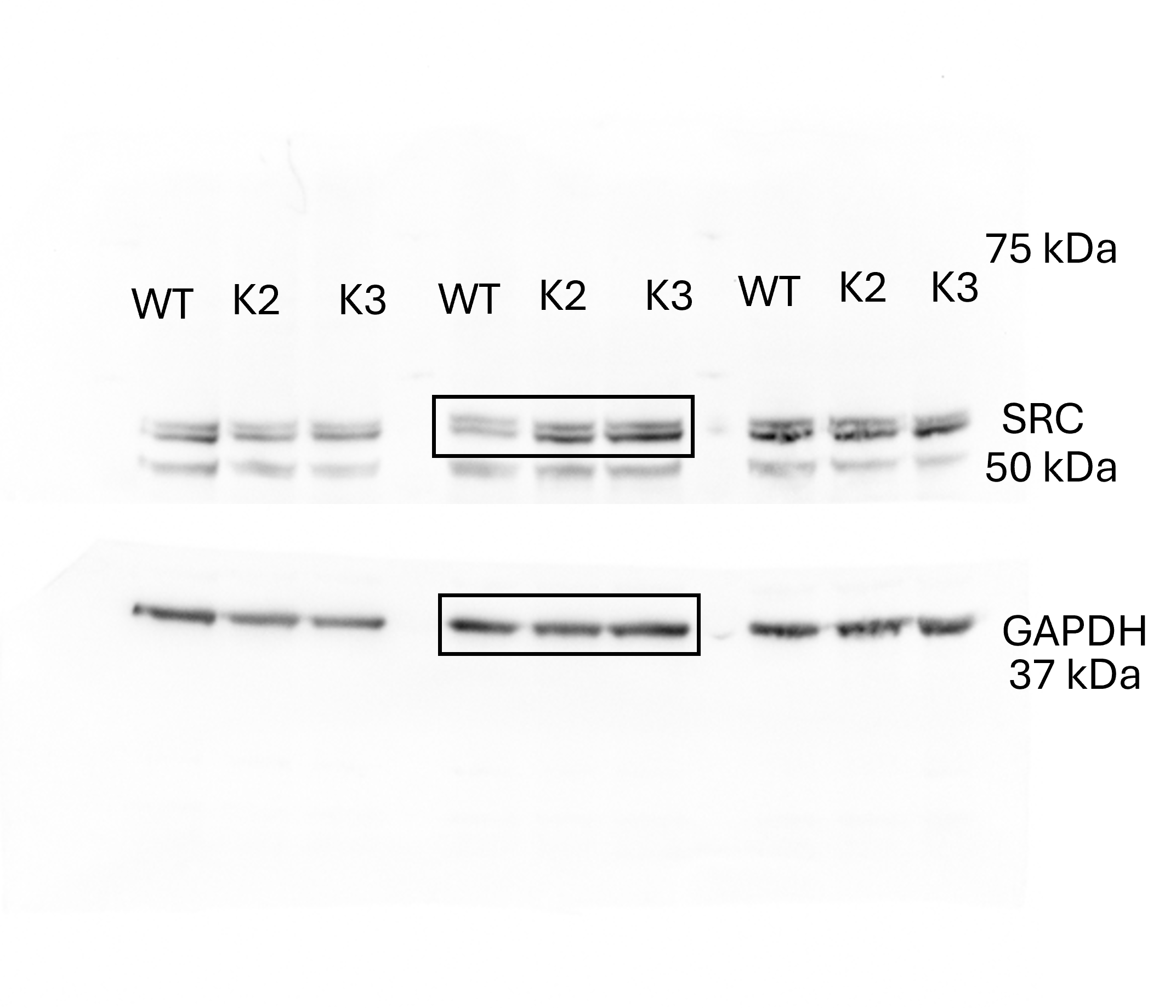

Supplement: Supplementary file 8 — Source data Fig. 4 [file 44318_2024_271_MOESM8_ESM.zip › Figure 4/4D_SRC_WB.tif]

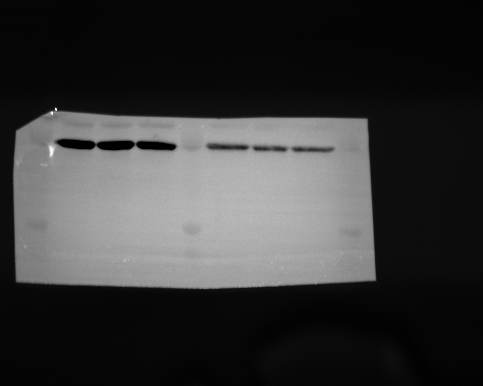

Supplement: Supplementary file 8 — Source data Fig. 4 [file 44318_2024_271_MOESM8_ESM.zip › Figure 4/06202024_GAPDH_2_SNX9_WT_K2_K3.tif]

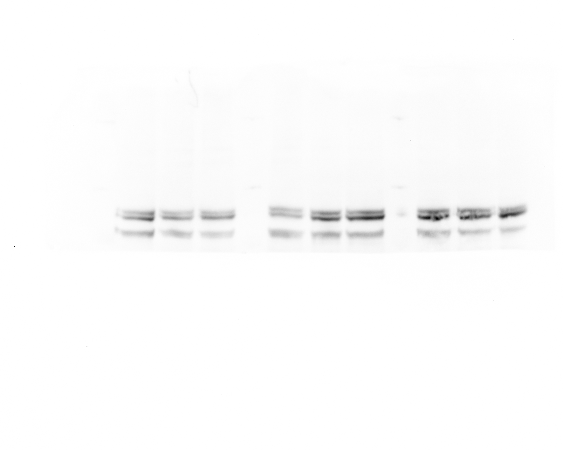

Supplement: Supplementary file 8 — Source data Fig. 4 [file 44318_2024_271_MOESM8_ESM.zip › Figure 4/05272024_SRC_2_1806_WT_K2_K3.tif]

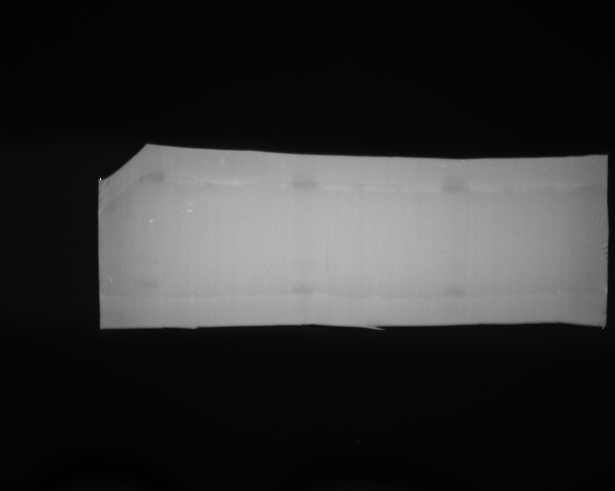

Supplement: Supplementary file 8 — Source data Fig. 4 [file 44318_2024_271_MOESM8_ESM.zip › Figure 4/05272024_Marker for GAPDH_SRC_WT,K2,K3.tif]

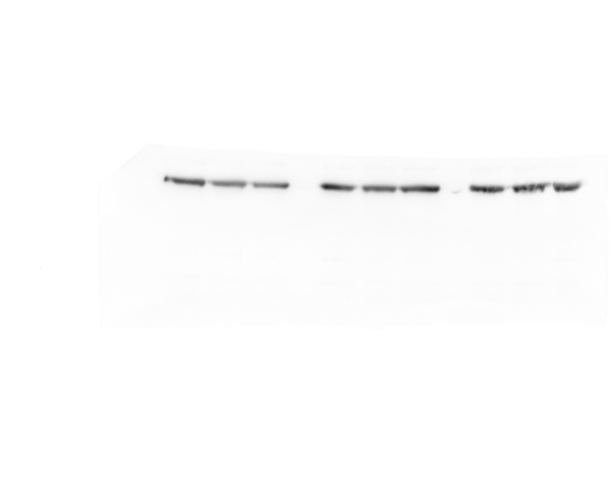

Supplement: Supplementary file 8 — Source data Fig. 4 [file 44318_2024_271_MOESM8_ESM.zip › Figure 4/05272024_GAPDH_2_1806_WT_K2_K3.tif]

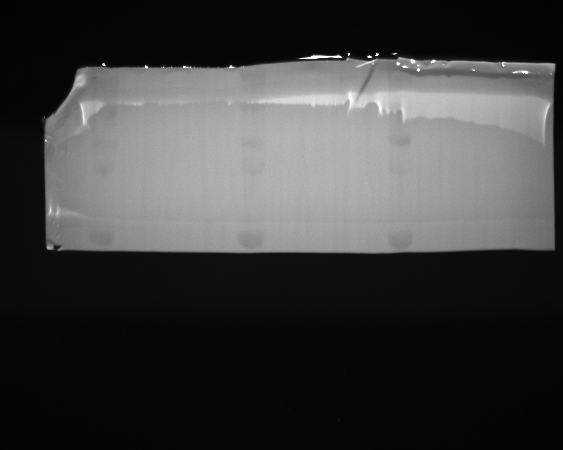

Supplement: Supplementary file 8 — Source data Fig. 4 [file 44318_2024_271_MOESM8_ESM.zip › Figure 4/05272024_Marker for SRC_WT,K2,K3.tif]

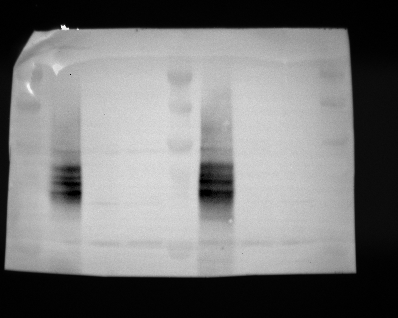

Supplement: Supplementary file 8 — Source data Fig. 4 [file 44318_2024_271_MOESM8_ESM.zip › Figure 4/06252024_ASCT2_SNX9BLOT_1806_WT_K2_K3.tif]

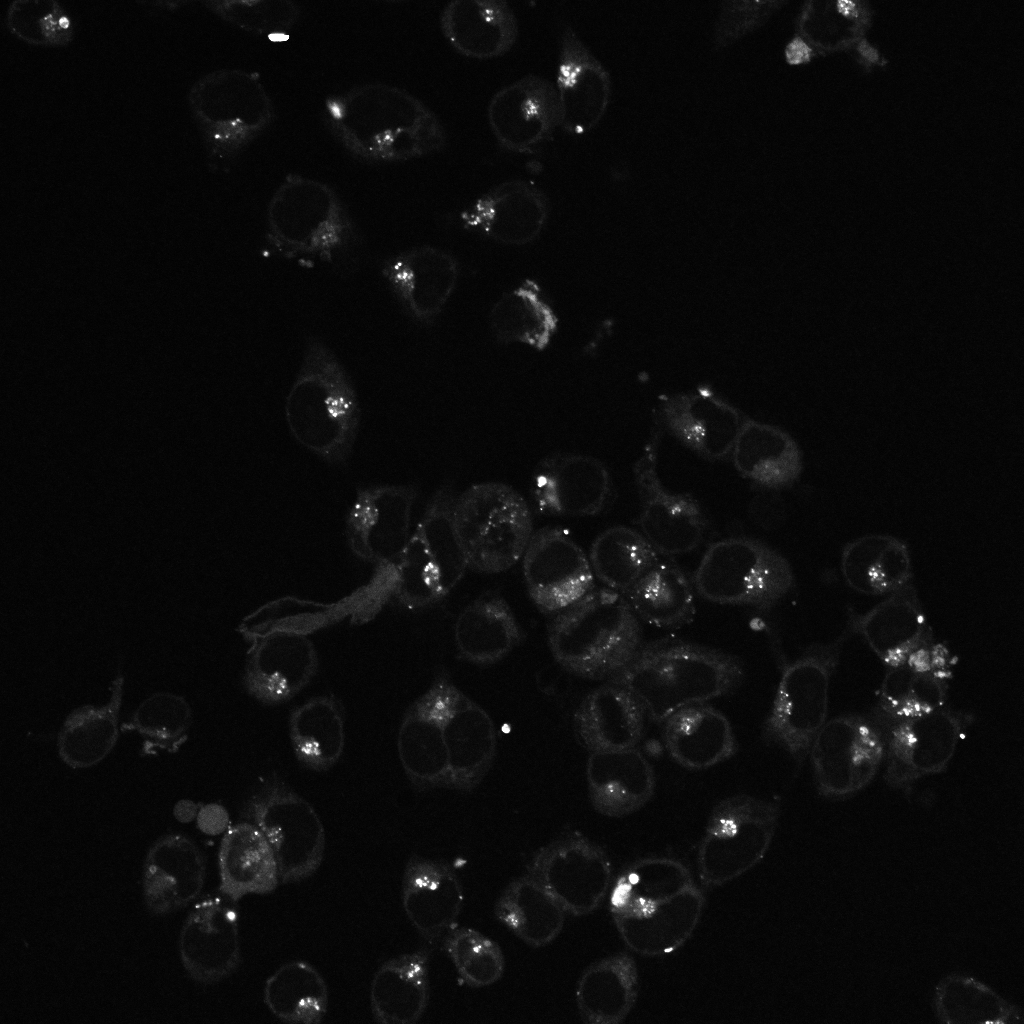

Supplement: Supplementary file 9 — Source data Fig. 5 [file 44318_2024_271_MOESM9_ESM.zip › Figure 5/5A/5A_K1.tif]

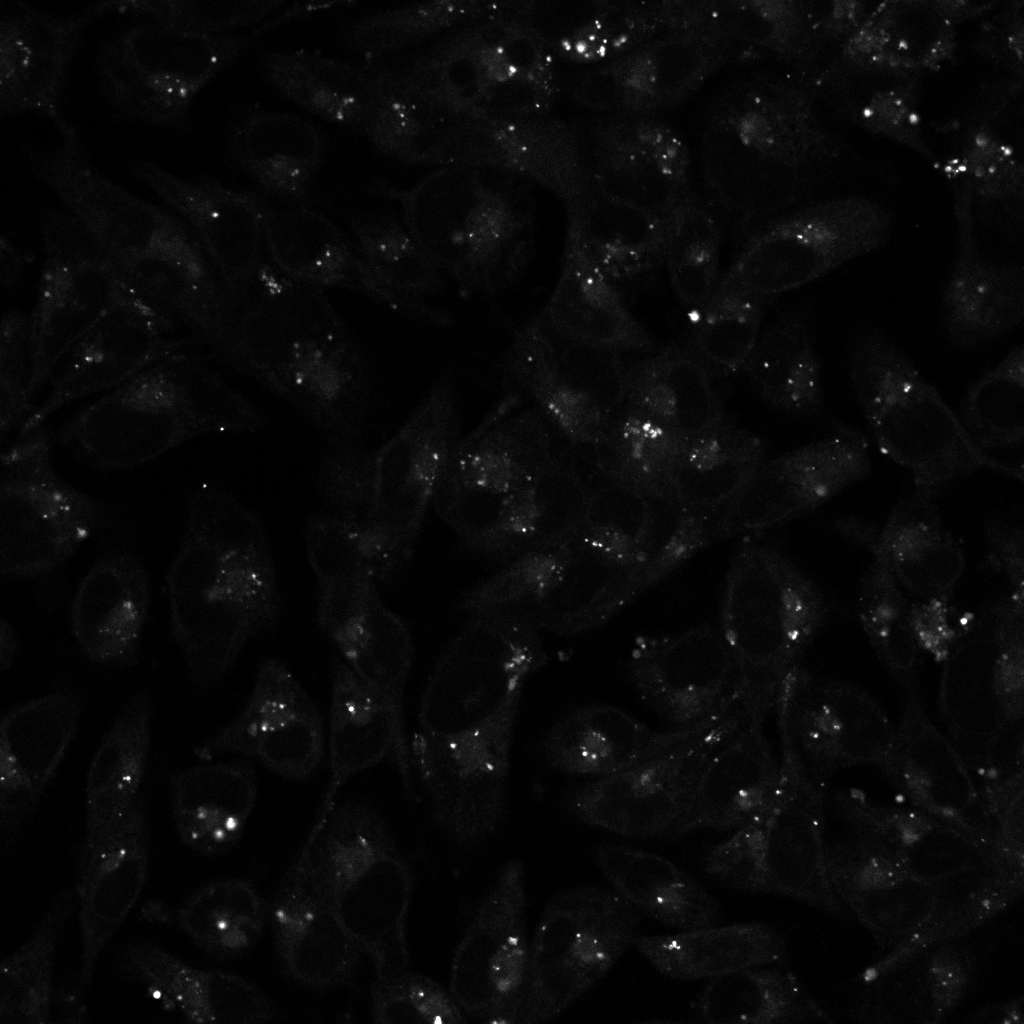

Supplement: Supplementary file 9 — Source data Fig. 5 [file 44318_2024_271_MOESM9_ESM.zip › Figure 5/5A/5A_HCC1806_shCTRL_Dox.tif]

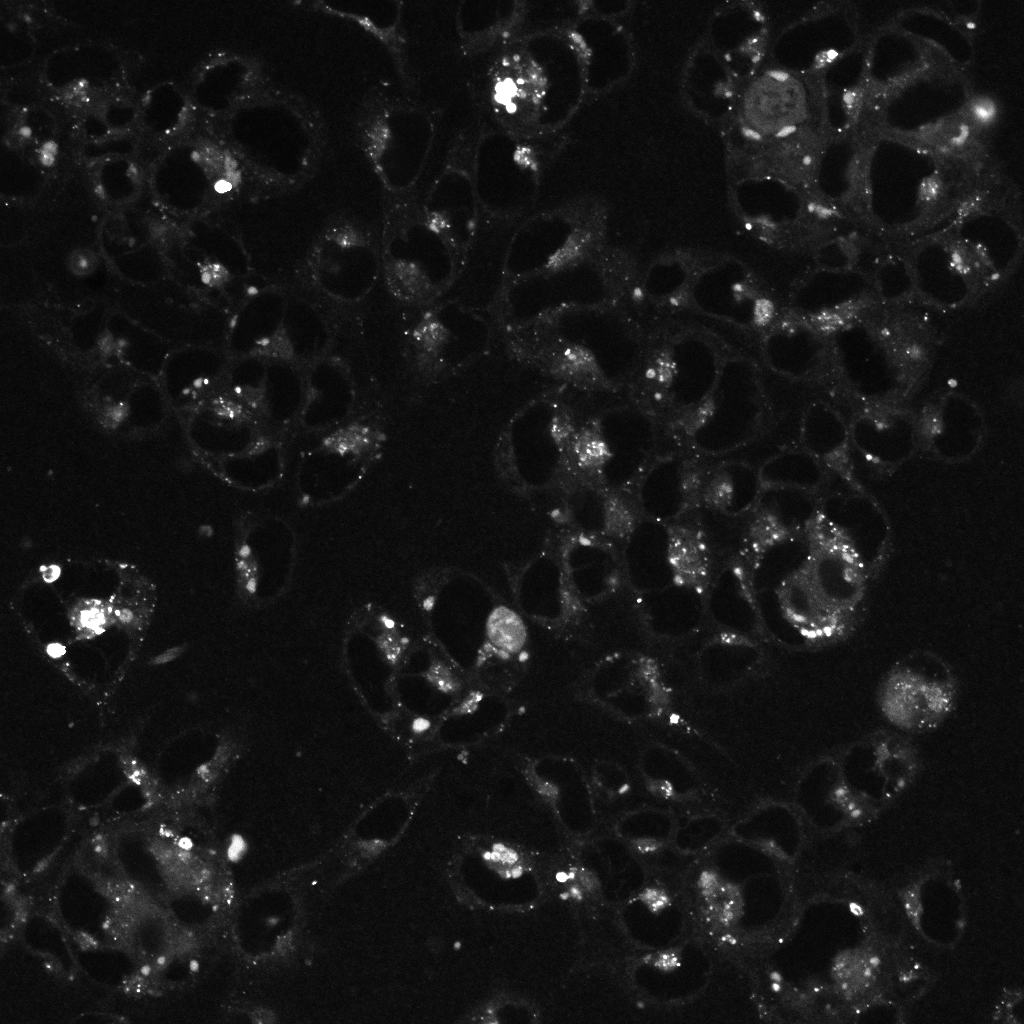

Supplement: Supplementary file 9 — Source data Fig. 5 [file 44318_2024_271_MOESM9_ESM.zip › Figure 5/5A/5A_K5.tif]

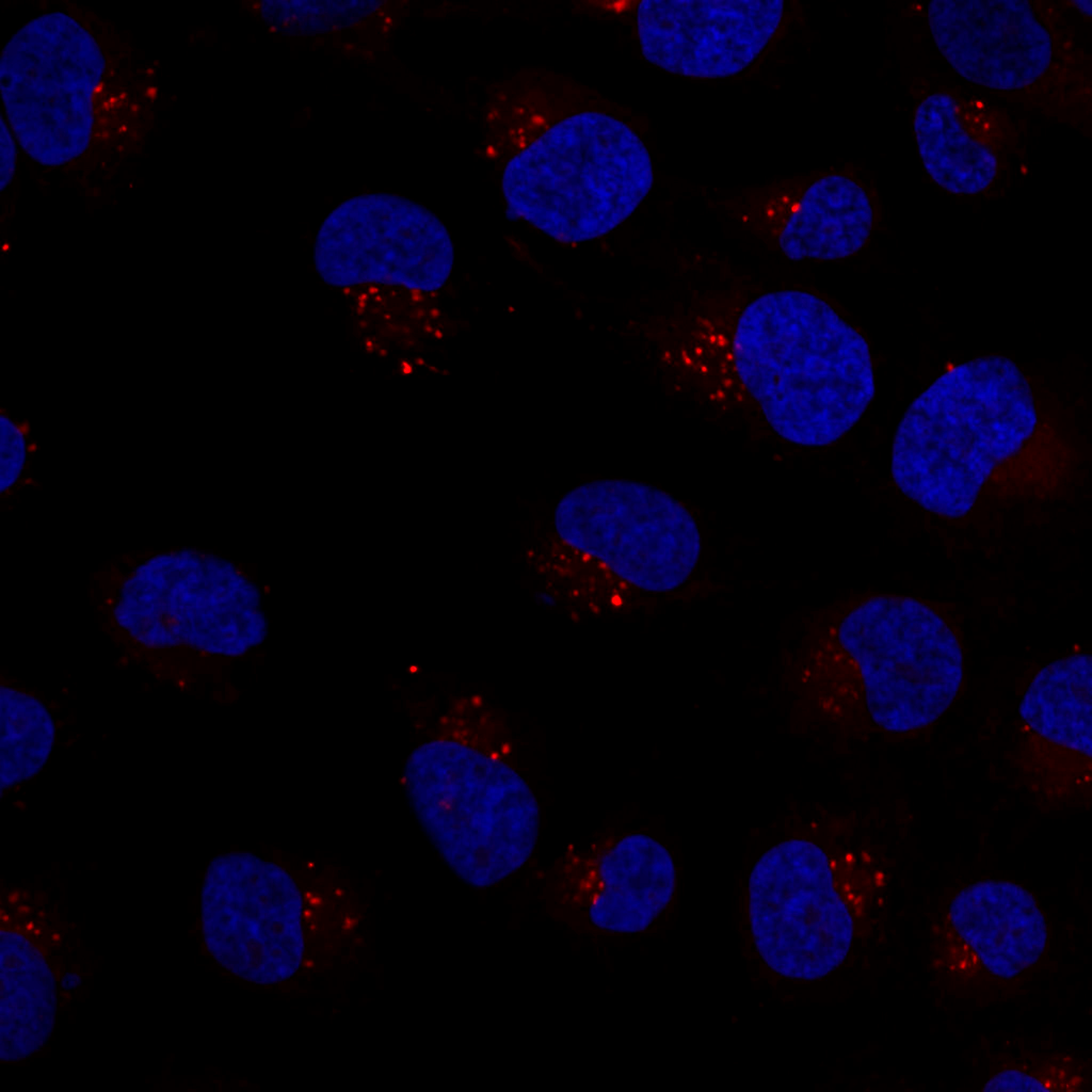

Supplement: Supplementary file 9 — Source data Fig. 5 [file 44318_2024_271_MOESM9_ESM.zip › Figure 5/5C/5C_1806_2_A2KO_20220120.tif]

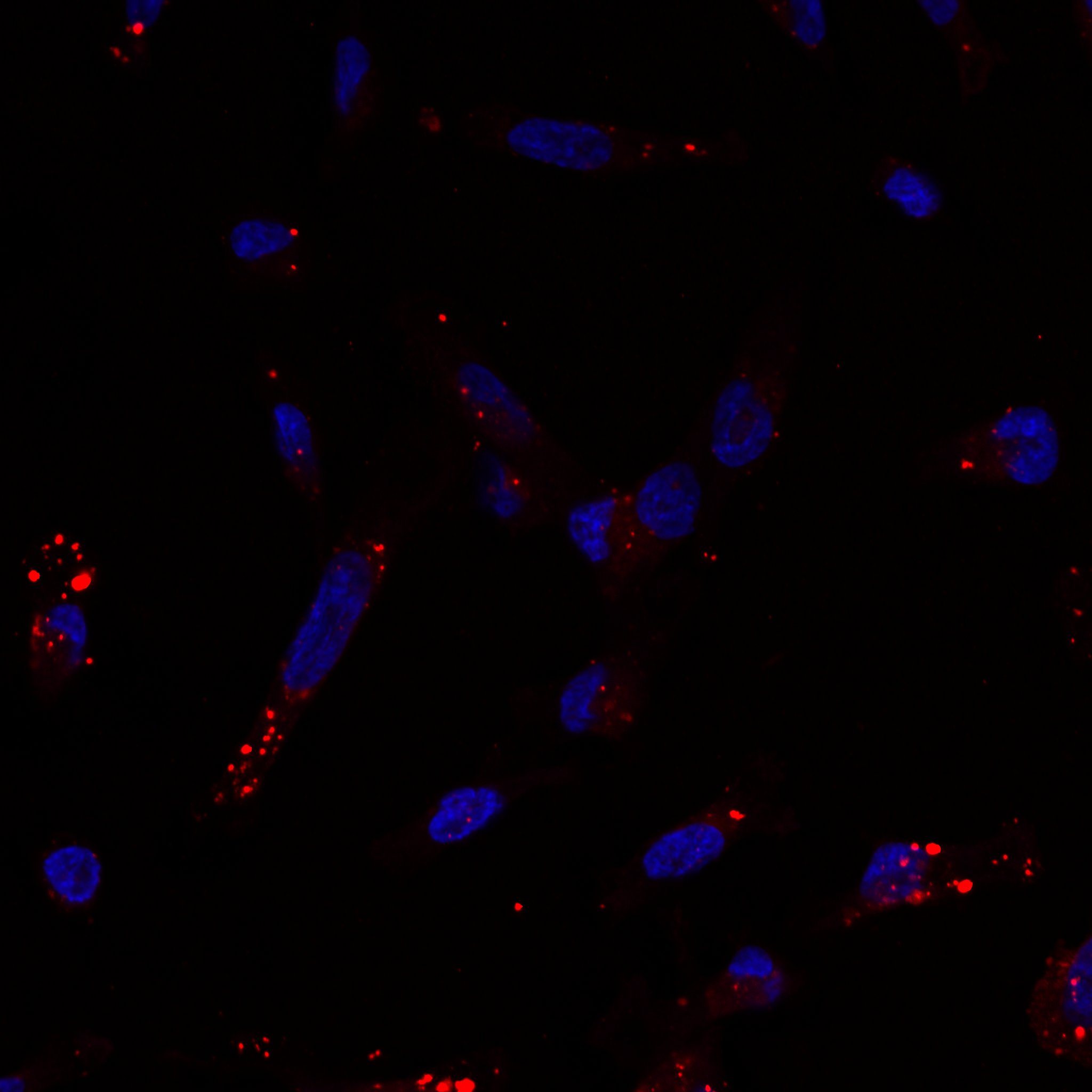

Supplement: Supplementary file 9 — Source data Fig. 5 [file 44318_2024_271_MOESM9_ESM.zip › Figure 5/5D/5D_231_A2KO_20220120.tif]

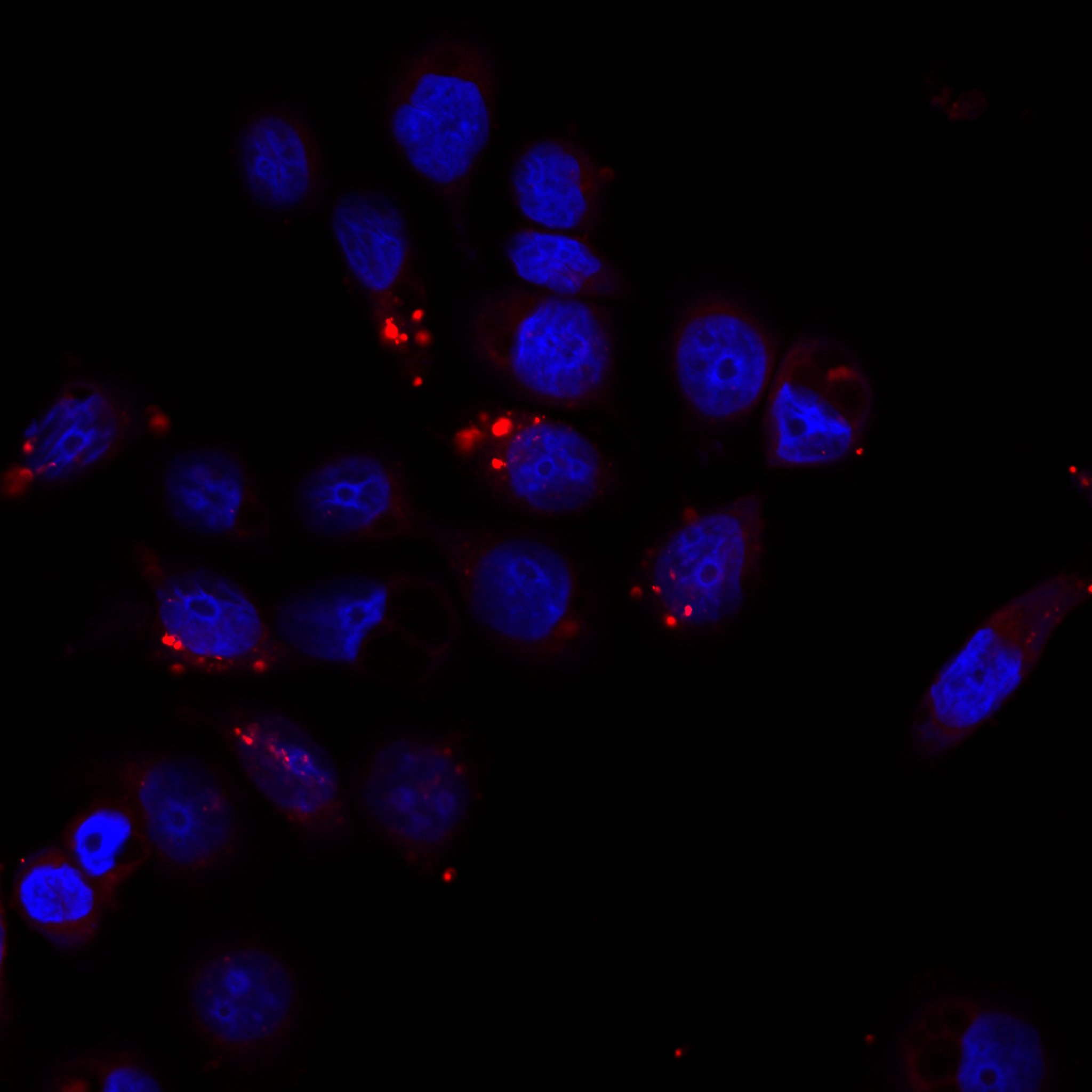

Supplement: Supplementary file 9 — Source data Fig. 5 [file 44318_2024_271_MOESM9_ESM.zip › Figure 5/5E/5E_468_NC_20220304.tif]

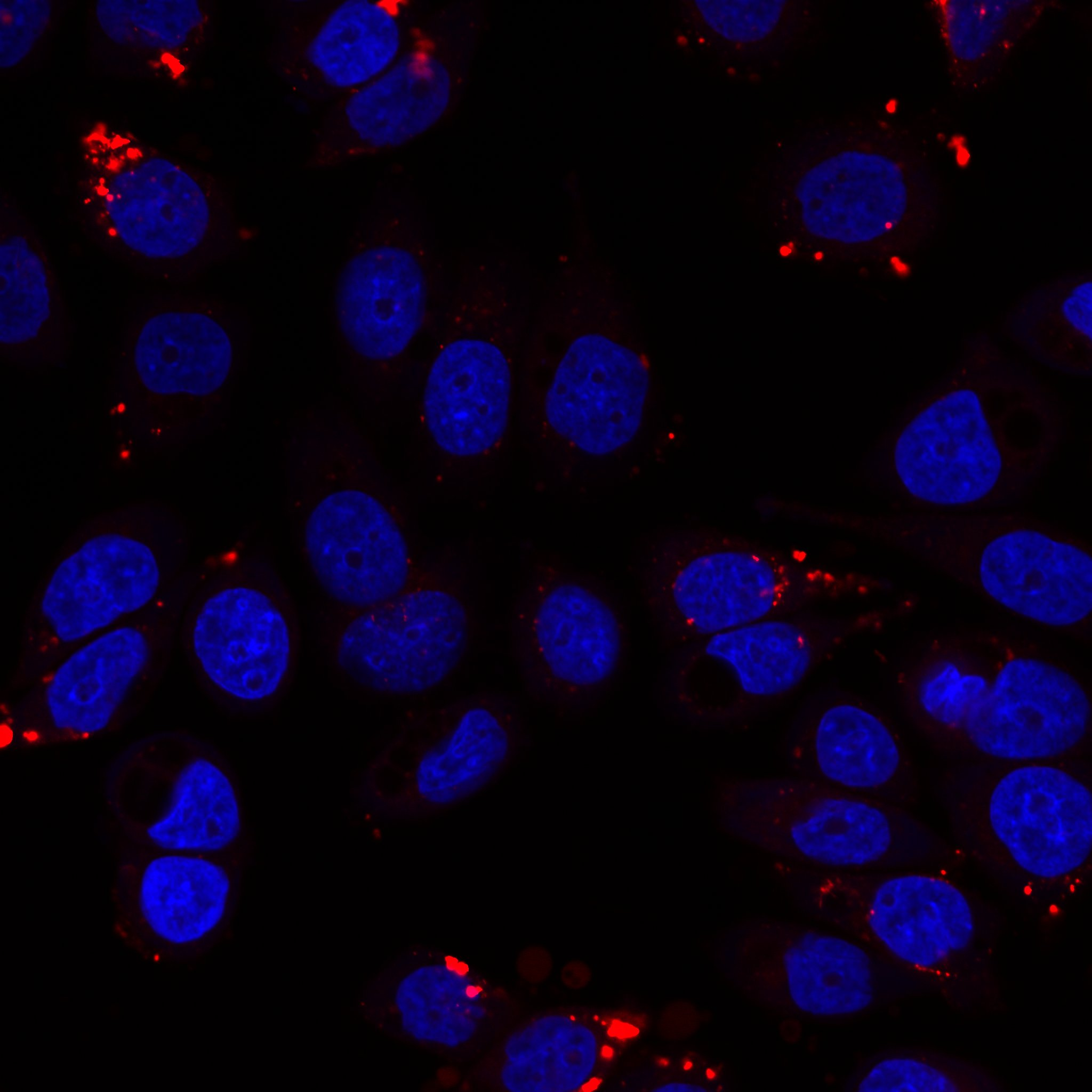

Supplement: Supplementary file 9 — Source data Fig. 5 [file 44318_2024_271_MOESM9_ESM.zip › Figure 5/5E/5E_468_A2KO_20220304.tif]

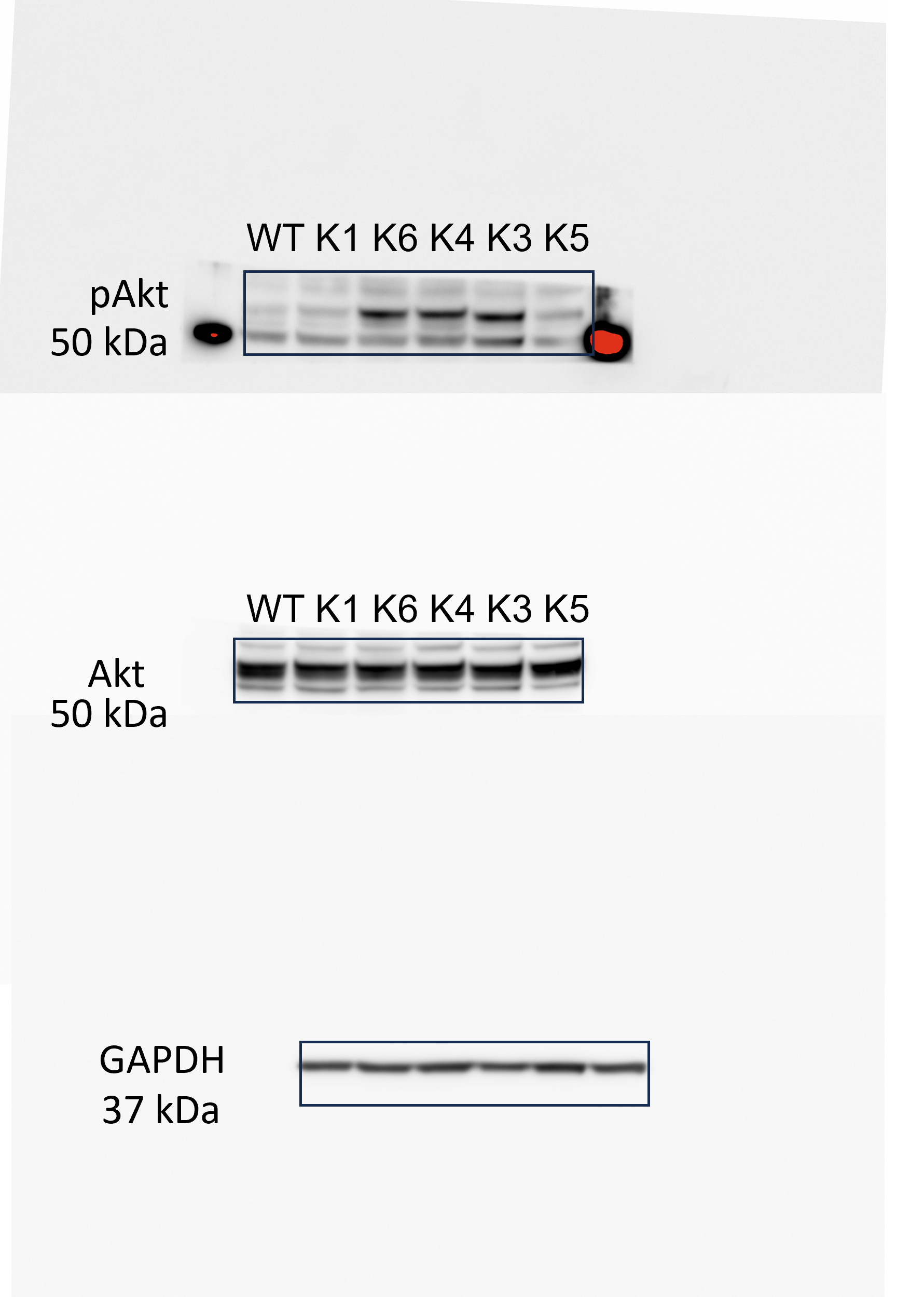

Supplement: Supplementary file 9 — Source data Fig. 5 [file 44318_2024_271_MOESM9_ESM.zip › Figure 5/5B/WB_pAkt, Akt,GAPDH.tif]

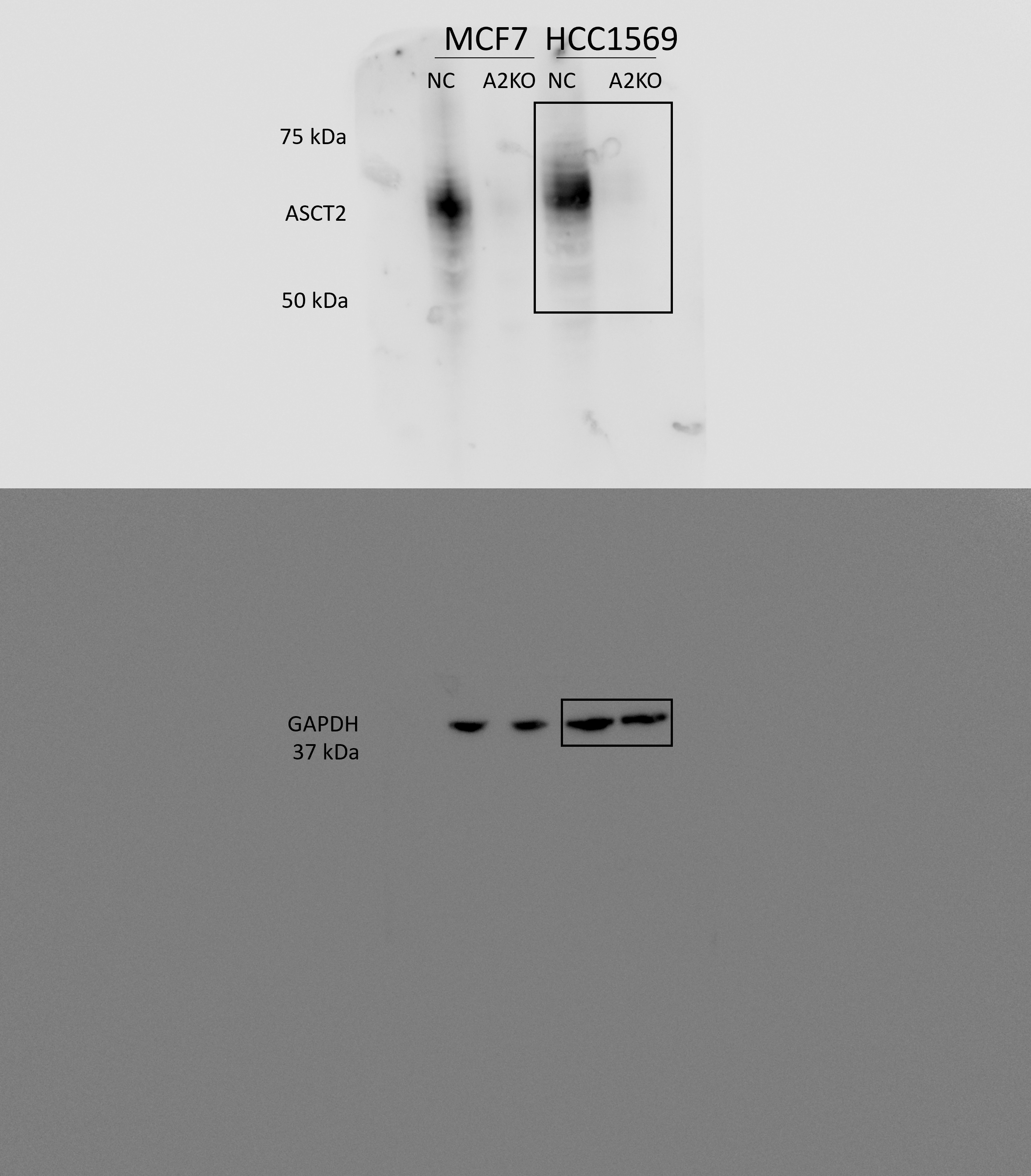

Supplement: Supplementary file 10 — Source data Fig. 6 [file 44318_2024_271_MOESM10_ESM.zip › Figure 6/6A/6A_HCC1569 WB.tif]

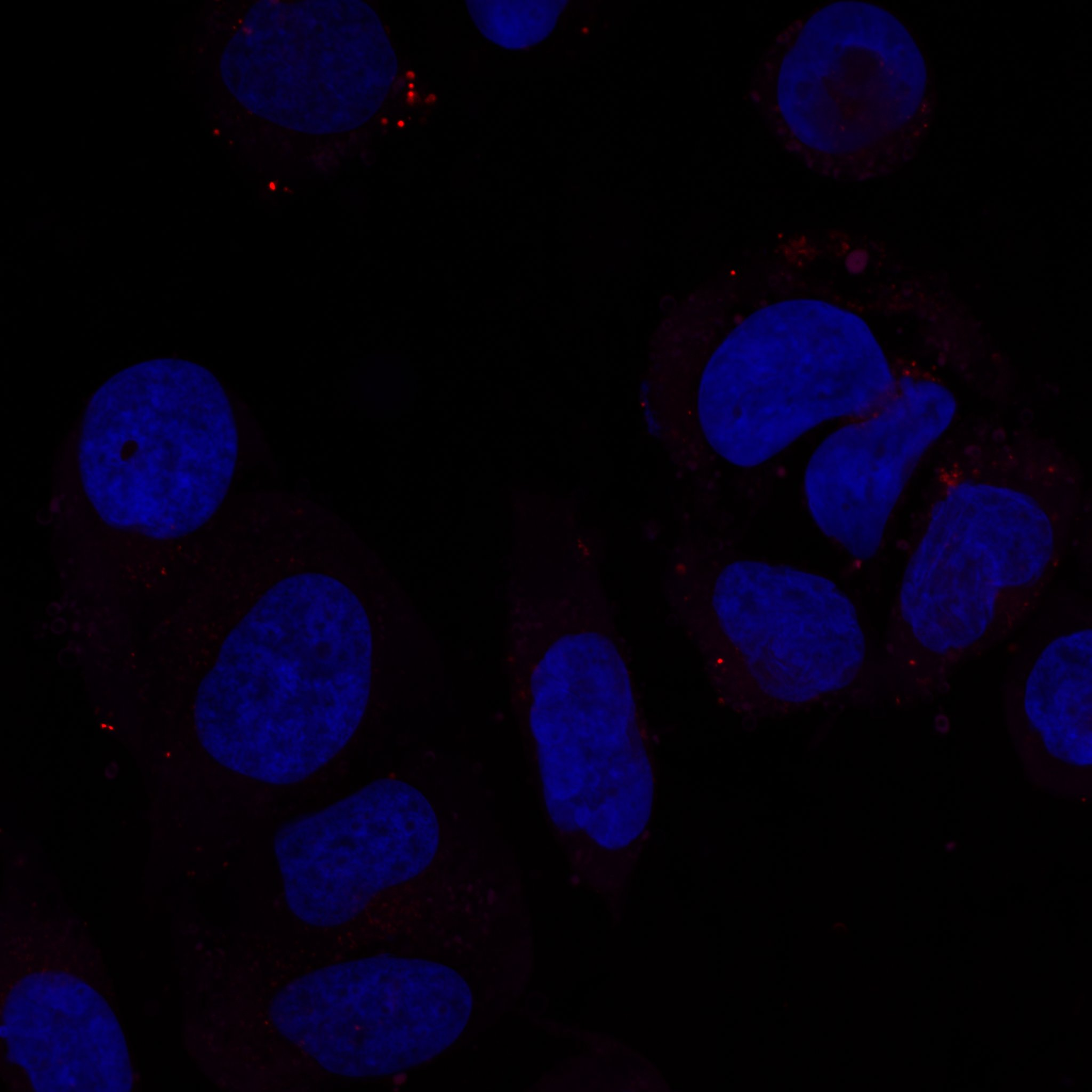

Supplement: Supplementary file 10 — Source data Fig. 6 [file 44318_2024_271_MOESM10_ESM.zip › Figure 6/6E/20220304_Image 1569_A2KO.tif]

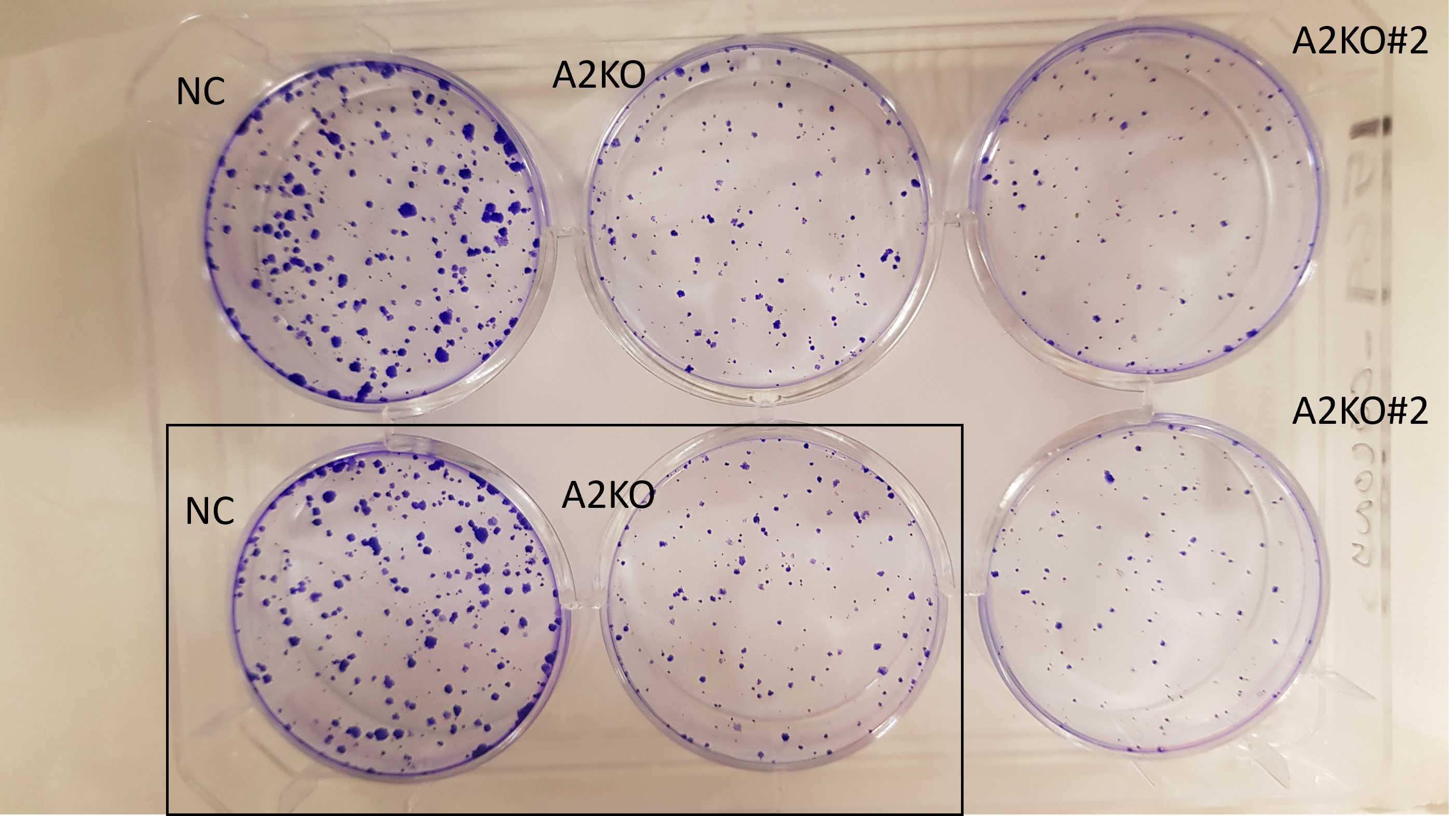

Supplement: Supplementary file 10 — Source data Fig. 6 [file 44318_2024_271_MOESM10_ESM.zip › Figure 6/6D/6D_HCC1569_colony formation assay.tif]
