# Supplementary material for: Macropinocytosis mediates resistance to loss of glutamine transport in triple-negative breast cancer
Source: EMBO J. 2024 Oct 17;43(23):5857–82. doi: 10.1038/s44318-024-00271-6 (PMC11611898; doi:10.1038/s44318-024-00271-6)
Supplement: Supplementary file 11 — Expanded View Figures [file 44318_2024_271_MOESM11_ESM.pdf]

# Expanded View Figures

**Figure EV1. ASCT2 knockout in additional breast cancer cell lines, relates to Fig. 1.**

(**A**) MTT assays for polyclonal HCC1806 NC and A2KO cells cultured in various glutamine concentrations with dialysed FBS (dFBS; 10% v/v). Mean ± SEM from three independent experiments performed in triplicate and analysed by two-way ANOVA; ns is not significant. (**B–M**) Polyclonal MDA-MB-468 (**B–E**), MCF7 (**F–I**) and T47D (**J–M**) NC and A2KO cell lines were generated by CRISPR. (**B**, **F**, **J**) Western blot for ASCT2 protein (CST #8057S, 60–80 kDa), with GAPDH protein (Abcam #ab8245, 37 kDa) as a loading control. (**C**, **G**, **K**) Uptake of 100 nM [$^3$H]-L-glutamine over 30 min, mean ± SEM from three independent experiments in triplicate and analysed by unpaired *T*-test where ****$p < 0.0001$. (**D**, **H**, **L**) CCK8 growth assays over 96 h, measured at timepoints indicated and normalised to the day 0 reading. Cells were seeded at a density of $5 \times 10^3$ (MDA-MB-468) and $1 \times 10^4$ (MCF7 and T47D) per well. Mean ± SEM from three independent experiments performed in triplicate and analysed by two-way ANOVA where ns is not significant. (**E**, **I**, **M**) Colony formation assay (CFA) in a six-well plate at $2.5 \times 10^3$ cells (MDA-MB-468) and $5 \times 10^3$ cells (MCF7 and T47D) per well, fixed and stained with 0.5% crystal violet after 12–14 days. Mean ± SEM from three independent experiments in triplicate and analysed by unpaired *T*-test where ****$p < 0.0001$ (**E**) and ns (**I**, **M**). (**N**) Schematic for *SLC1A5* gene (ASCT2) and CRISPR guide RNA (red) targeting exon 4. The image is not to scale. Polyclonal HCC1806 NC#2 and A2KO#2 cells generated with exon 4 guide RNA and assessed for (**O**) ASCT2 expression with GAPDH as control by western blot, (**P**) [$^3$H]-L-glutamine uptake over 30 min, mean ± SEM from three independent experiments in triplicate and analysed by unpaired *T*-test where ****$p < 0.0001$ and (**Q**) cell growth (relative to day 0) over 96 h by CCK8 assay, data are mean ± SEM from three independent experiments performed in triplicate and analysed by two-way ANOVA where ns is not significant.

 © The Author(s)

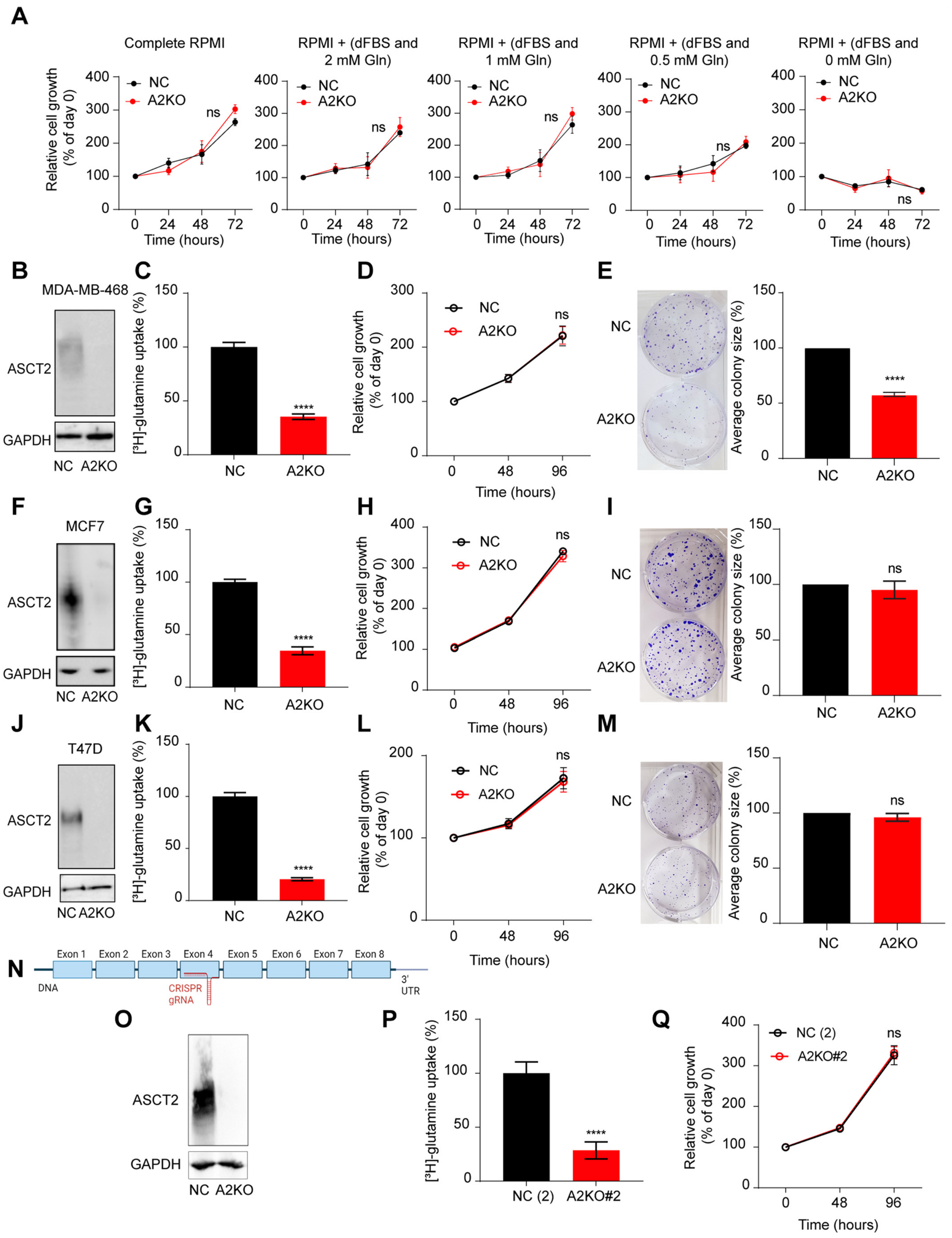
A
Complete RPMI
RPMI + (dFBS and 2 mM Gln)
RPMI + (dFBS and 1 mM Gln)
RPMI + (dFBS and 0.5 mM Gln)
RPMI + (dFBS and 0 mM Gln)
Relative cell growth (% of day 0)
NC
A2KO
ns
Time (hours)
B
MDA-MB-468
ASCT2
GAPDH
NC A2KO
C
[³H]-glutamine uptake (%)
****
D
Relative cell growth (% of day 0)
E
Average colony size (%)
F
MCF7
G
H
I
J
T47D
K
L
M
N
Exon 1
Exon 2
Exon 3
Exon 4
Exon 5
Exon 6
Exon 7
Exon 8
DNA
CRISPR gRNA
3' UTR
O
P
NC (2)
A2KO#2
Q
NC (2)
A2KO#2

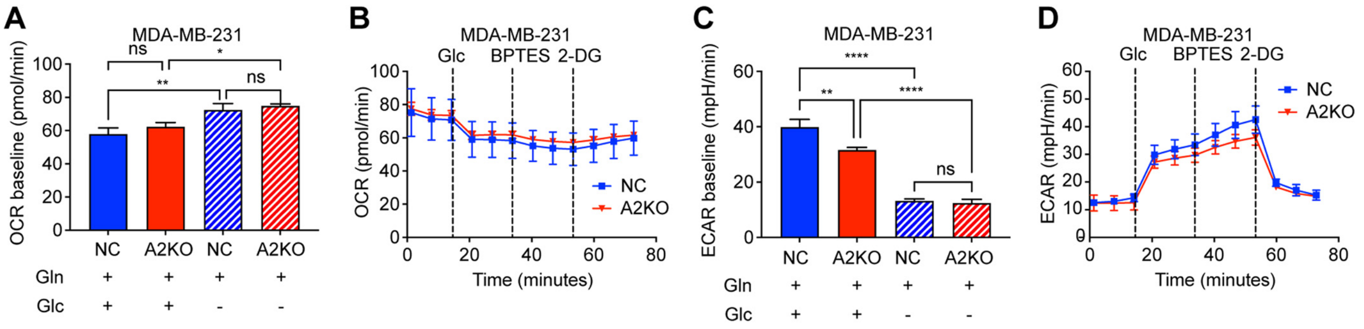


**Figure EV2. Rate of glycolysis appears to be lower in MDA-MD-231 A2KO cells, relates to Fig. 2.**

(**A**) OCR was measured in MDA-MB-231 cells over 14 min in complete media or media lacking glucose, $^{**}p = 0.0067$, $^{*}p = 0.0230$. (**B**) Seahorse assay was carried out in MDA-MB-231 cells, and OCR was measured in glucose-free media, with sequential acute injection of 25 mM glucose, followed by 3 μM BPTES (glutaminase inhibitor) and finally 50 mM 2-DG (glycolysis inhibitor). (**C**) ECAR was measured in MDA-MB-231 cells over 14 min in complete media or media lacking glucose, $^{**}p = 0.0044$, $^{****}p < 0.0001$. (**D**) Seahorse assay was carried out in MDA-MB-231 cells, and ECAR was measured in glucose-free media, with a sequential acute injection of 25 mM glucose, followed by 3 μM BPTES (glutaminase inhibitor) and finally 50 mM 2-DG (glycolysis inhibitor). Data are mean ± SEM from three independent experiments in triplicate, analysed by one-way ANOVA with Šídák's multiple comparisons test (ns not significant).

 © The Author(s)

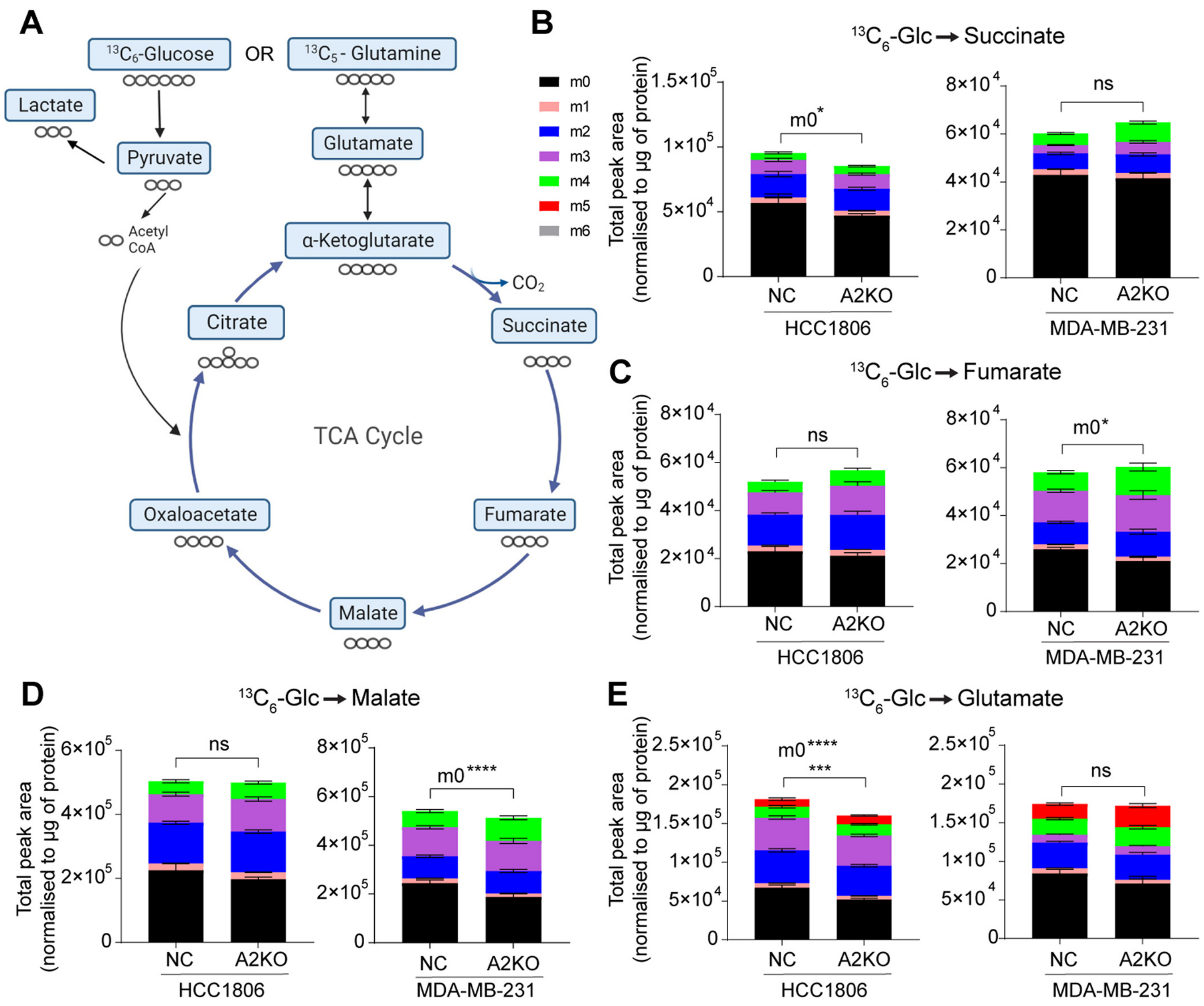


**Figure EV3. TCA metabolite levels remain predominantly unchanged in A2KO cells, relates to Fig. 3.**

(**A**) Schematic for tracing $^{13}C_5$-glutamine or $^{13}C_6$-glucose carbons through glycolysis and the TCA cycle in polyclonal HCC1806 and MDA-MB-231 NC and A2KO cells. (**B–E**) Total peak area of $^{13}C_6$-glucose ($^{13}C_6$-Glc) derived TCA metabolites succinate (**B**), fumarate (**C**) and malate (**D**) as well as glutamate (**E**) were detected by LC-MS at 24 h. *p* values for differences between NC and A2KO cells for (**B**) succinate in HCC1806 m0 $*p = 0.0344$ and MDA-MB-231 not significant (ns) for all, (**D**) fumarate in HCC1806 ns for all and MDA-MB-231 m0 $*p = 0.0111$, (**D**) malate in HCC1806 ns for all and MDA-MB-231 m0 $****p < 0.0001$, (**E**) glutamate in HCC1806 $***p = 0.0006$, m0 $****p < 0.0001$ and MDA-MB-231 ns for all. Mass of the unlabelled metabolite ($^{12}C = m0$), which changes with integration with $^{13}C$-labelled carbons, where (m#) indicates a metabolite with # of carbons labelled with $^{13}C$. Isotopologue (m0) denotes that all carbons of the metabolite are $^{12}C$, and the metabolite is unlabelled. Isotopologue (m6) signifies that six carbons are $^{13}C$ isotopes ($^{13}C_6$-Glc). The total peak area was normalised to µg of protein for each sample. Error bars are mean ± SEM from three independent experiments performed in duplicate analysed by two-way ANOVA. Where isotopologues are listed with asterisks, the unlisted isotopologues are ns.

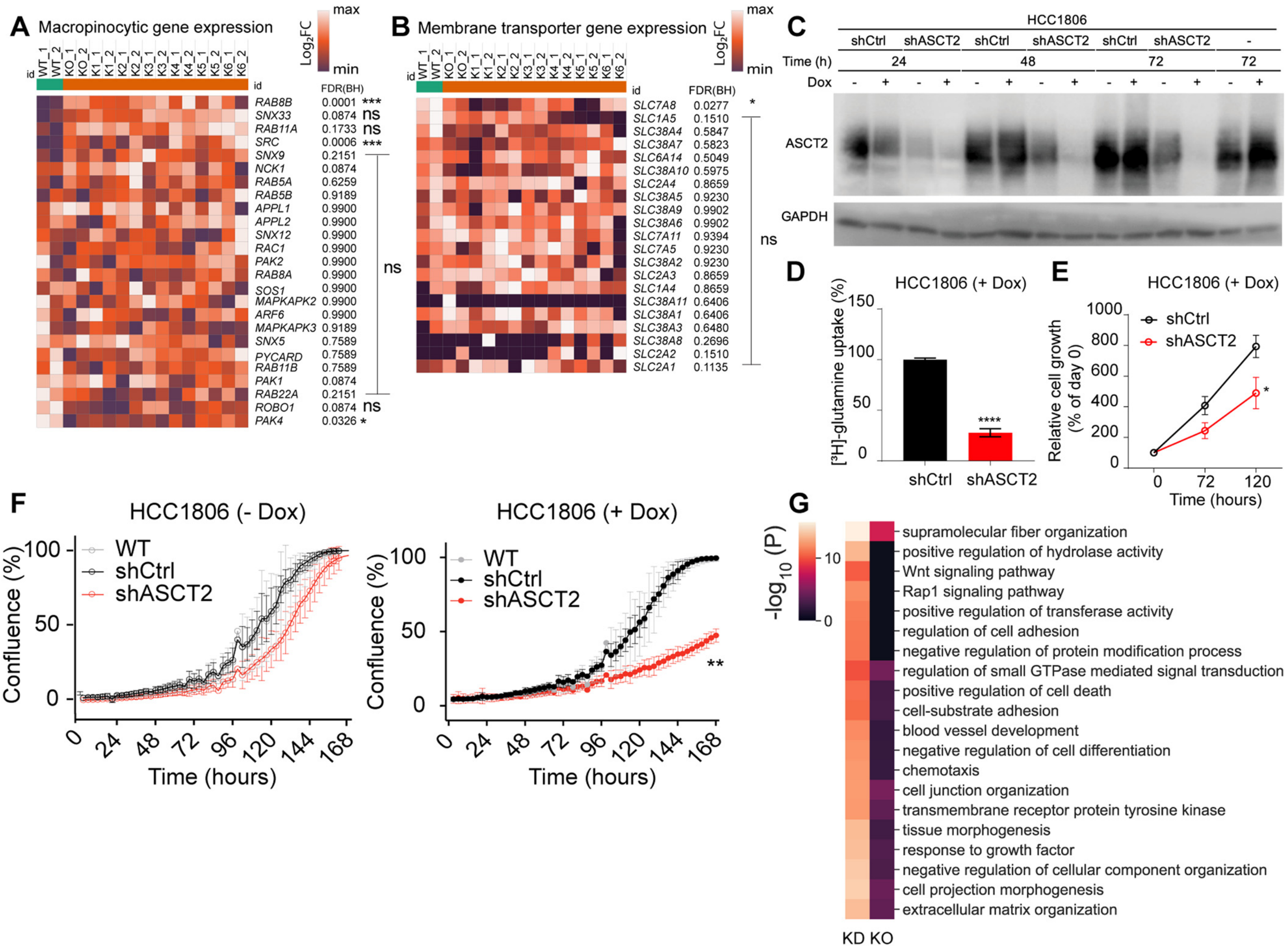


**Figure EV4. Macropinocytic and transporter mRNA expression heatmaps and effects of ASCT2 shRNA knockdown in HCC1806, relates to Fig. 4.**

(**A**, **B**) Differentially expressed genes from RNA-seq relevant to macropinocytosis and membrane transport in HCC1806 cells. All data were normalised and log2-transformed, and a pseudocount of 0.1 was applied in DESeq2 v1.26.0 software. DESeq2 was also used to select differentially expressed genes ('DEGs') HCC1806 WT and each HCC1806 ASCT2 knockout clones (KO and K1–K6) ($n = 2$ biological replicates). A relative colour scheme is used for heatmaps (**A**, **C**, **E**, **F**) where minimum (dark brown) and maximum (beige) values in each row are converted to colours (https://software.broadinstitute.org/morpheus/). (**C**) Western blot of ASCT2 protein (CST #8057S, 60–80 kDa) and GAPDH protein (Abcam #ab8245, 37 kDa) as a loading control in HCC1806 cell lines stably transfected with shCtrl and shASCT2 after 72 h pre-incubation in dox. Cells were maintained in tetracycline-free media ±1 µg/mL dox (as indicated by -/+ dox). (**D**) Uptake of 100 nM [$^3$H]-glutamine in HCC1806 cell lines over 30 min, after 72 h pre-incubation in dox, mean ± SEM from three independent experiments in triplicate and analysed by unpaired *t*-test where $^{****}p < 0.0001$. (**E**) CCK8 assay of HCC1806 cell lines measured at timepoints indicated ± dox. Cells were seeded at a density of $1 \times 10^3$ cells per well and grown in dox for the duration of the CCK8 assay where mean ± SEM from three independent experiments performed in triplicate and analysed by two-way ANOVA where $^{*}p = 0.0428$. (**F**) Growth of HCC1806 cell lines measured by live cell imaging (IncuCyte® S3) over 168 h, at 3 h intervals. Cells were seeded at a density of $1 \times 10^3$ cells per well and grown ± dox for the duration of the assay, data are mean ± SEM from three independent experiments performed in triplicate and analysed by two-way ANOVA where $^{**}p = 0.0034$. (**G**) Heatmap of concordantly enriched pathways comparing ASCT2 knockdown (KD) and ASCT2 KO (KO), where *P* indicates hypergeometric *p* value for enrichment computed in Metascape.

 © The Author(s)

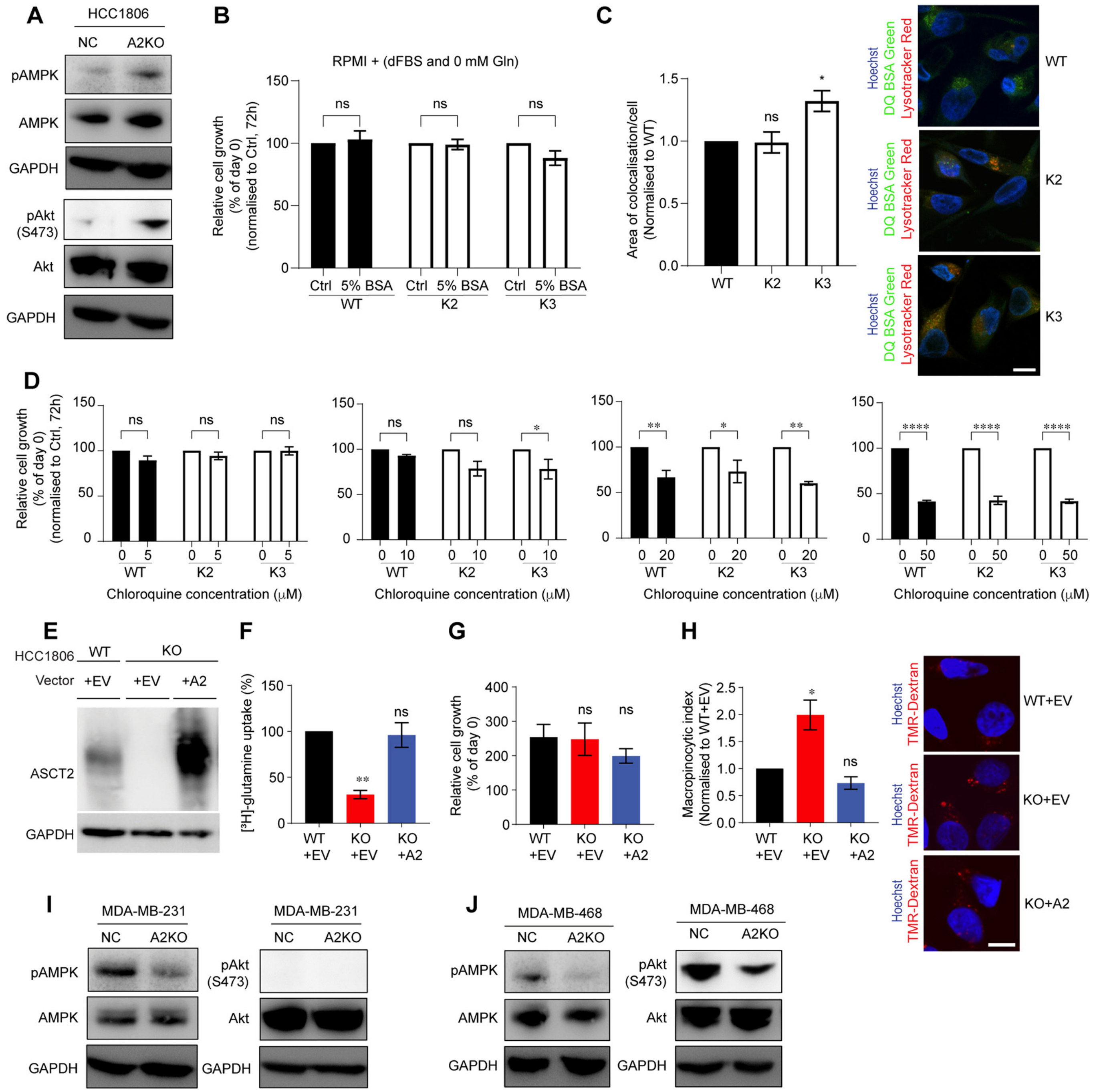
A
HCC1806
NC
A2KO
pAMPK
AMPK
GAPDH
pAkt (S473)
Akt
GAPDH
B
RPMI + (dFBS and 0 mM Gln)
Relative cell growth (% of day 0) (normalised to Ctrl, 72h)
ns
Ctrl
5% BSA
WT
K2
K3
C
Area of colocalisation/cell (Normalised to WT)
*
WT
K2
K3
Hoechst
DQ BSA Green
Lysotracker Red
D
Chloroquine concentration (μM)
ns
*
**
****
E
HCC1806
WT
KO
Vector
+EV
+A2
ASCT2
GAPDH
F
[3H]-glutamine uptake (%)
WT +EV
KO +EV
KO +A2
G
Relative cell growth (% of day 0)
H
Macropinocytic index (Normalised to WT+EV)
Hoechst
TMR-Dextran
WT+EV
KO+EV
KO+A2
I
MDA-MB-231
NC
A2KO
pAMPK
AMPK
GAPDH
pAkt (S473)
Akt
J
MDA-MB-468

**Figure EV5. AMPK and Akt phosphorylation is higher in HCC1806 A2KO cells, relates to Fig. 5.**

(**A**) Western blot of pAMPK protein (CST #2535, 62 kDa), AMPK (CST #5831, 62 kDa) and GAPDH protein (Abcam #ab8245, 37 kDa) and pAKT (S473) protein (CST #9271, 60 kDa), Akt (CST #9272, 60 kDa) and GAPDH protein (Abcam #ab8245, 37 kDa) as a loading control in polyclonal HCC1806 NC and A2KO cell lines. Representative image from two to three independent repeats is shown. (**B**) CCK8 assays for HCC1806 WT, K2 and K3 ASCT2 KO cell lines cultured in low glutamine conditions with dialysed FBS (dFBS; 10% v/v) ±5% fatty acid-free BSA. Mean ± SEM from four independent experiments in triplicate and analysed by two-way ANOVA where ns is not significant. (**C**) Area of DQ-BSA Green and Lysotracker Red co-localisation (yellow) quantified per cell in HCC1806 WT, K2 and K3 ASCT2 KO cell lines. Representative images, from three independent repeats of DQ-BSA Green and Lysotracker Red co-localisation, where nuclei were visualised with Hoescht fluorescent stain (blue). The scale bar in white is equivalent to 8 μM. Mean ± SEM of indices calculated from >30 cells (3–4 fields of view) across three independent experiments. Asterisks indicate *p* values, ns not significant, $^{*}p = 0.03$ from a one-way ANOVA. (**D**) CCK8 assays for HCC1806 WT, K2 and K3 ASCT2 KO cell lines cultured in 2 mM glutamine with dialysed FBS (dFBS; 10% v/v) ± water control/chloroquine (CQ) (5–50 μM). Mean ± SEM from three independent experiments in triplicate and analysed by two-way ANOVA where ns is not significant, 10 μM CQ $^{*}p = 0.0487$ for K3, 20 μM CQ $^{**}p = 0.0059$ for WT, $^{*}p = 0.0239$ for K2, $^{**}p = 0.0015$ for K3 and 50 μM CQ $^{****}p < 0.0001$ for WT, K2 and K3. (**E**) Western blot of ASCT2 protein (CST #8057S, 60–80 kDa) and GAPDH protein (Abcam #ab8245, 37 kDa) as a loading control in HCC1806 WT cell line transduced with empty vector (EV) or ASCT2 KO cells transduced with EV or ASCT2 (A2; rescue). (**F**) Uptake of 100 nM [$^{3}$H]-L-glutamine in HCC1806 cell lines over 30 min. Mean ± SEM from three independent experiments in triplicate where asterisks indicate *p* value from a one-way ANOVA where $^{**}p = 0.002$ and ns is not significant. (**G**) CCK8 assay of HCC1806 cell lines measured at 96 h, mean ± SEM from three independent experiments done in triplicate and analysed by two-way ANOVA where ns is not significant. (**H**) Uptake of TMR-dextran (red) in HCC1806 WT + EV, KO + EV and KO + A2. Nuclei were visualised with Hoescht fluorescent stain (blue). The scale bar in white is equivalent to 10 μM. Quantification of macropinocytic index in HCC1806 cell lines is macropinocytosis/cell (area of macropinosomes/number of cells in field). Representative images are shown from three independent repeats. Mean ± SEM of indices calculated from >30 cells (3–4 fields of view) across three independent experiments. Asterisks indicate *p* value from a one-way ANOVA where $^{*}p = 0.01$ and ns is not significant). (**I, J**) Western blot of pAMPK protein (CST #2535, 62 kDa), AMPK (CST #5831, 62 kDa) and GAPDH protein (Abcam #ab8245, 37 kDa) and pAKT (S473) protein (CST #9271, 60 kDa), Akt (CST #9272, 60 kDa) and GAPDH protein (Abcam #ab8245, 37 kDa) as a loading control in polyclonal MDA-MB-231 (**I**) and MDA-MB-468 (**J**) NC and A2KO cell lines. Representative image from two to three independent repeats is shown.


© The Author(s)
